# Supplementary material for: Combined Point-of-Care Nucleic Acid and Antibody Testing for SARS-CoV-2 following Emergence of D614G Spike Variant
Source: Cell Rep Med. 2020 Sep 1;1(6):100099. doi: 10.1016/j.xcrm.2020.100099 (PMC7462534; doi:10.1016/j.xcrm.2020.100099)
Supplement: Document S1. Figures S1–S4 and Tables S1 and S2 [file mmc1.pdf]

Cell Reports Medicine, Volume *1*

Supplemental Information

Combined Point-of-Care Nucleic Acid and Antibody Testing for SARS-CoV-2 following Emergence of D614G Spike Variant

Petra Mlcochova, Dami Collier, Allyson Ritchie, Sonny M. Assennato, Myra Hosmillo, Neha Goel, Bo Meng, Krishna Chatterjee, Vivien Mendoza, Nigel Temperton, Leo Kiss, Leo C. James, Katarzyna A. Ciazynska, Xiaoli Xiong, John A.G. Briggs, James A. Nathan, Federica Mescia, Laura Bergamaschi, Hongyi Zhang, Petros Barmpounakis, Nikos Demeris, Richard Skells, Paul A. Lyons, John Bradley, Steven Baker, Jean Pierre Allain, Kenneth G.C. Smith, Rachel Bousfield, Michael Wilson, Dominic Sparkes, Glenn Amoroso, Effrosyni Gkrania-Klotsas, Susie Hardwick, Adrian Boyle, Ian Goodfellow, Ravindra K. Gupta, and The Cambridge Institute of Therapeutic Immunology and Infectious Disease-National Institute of Health Research (CITIID-NIHR) COVID BioResource Collaboration

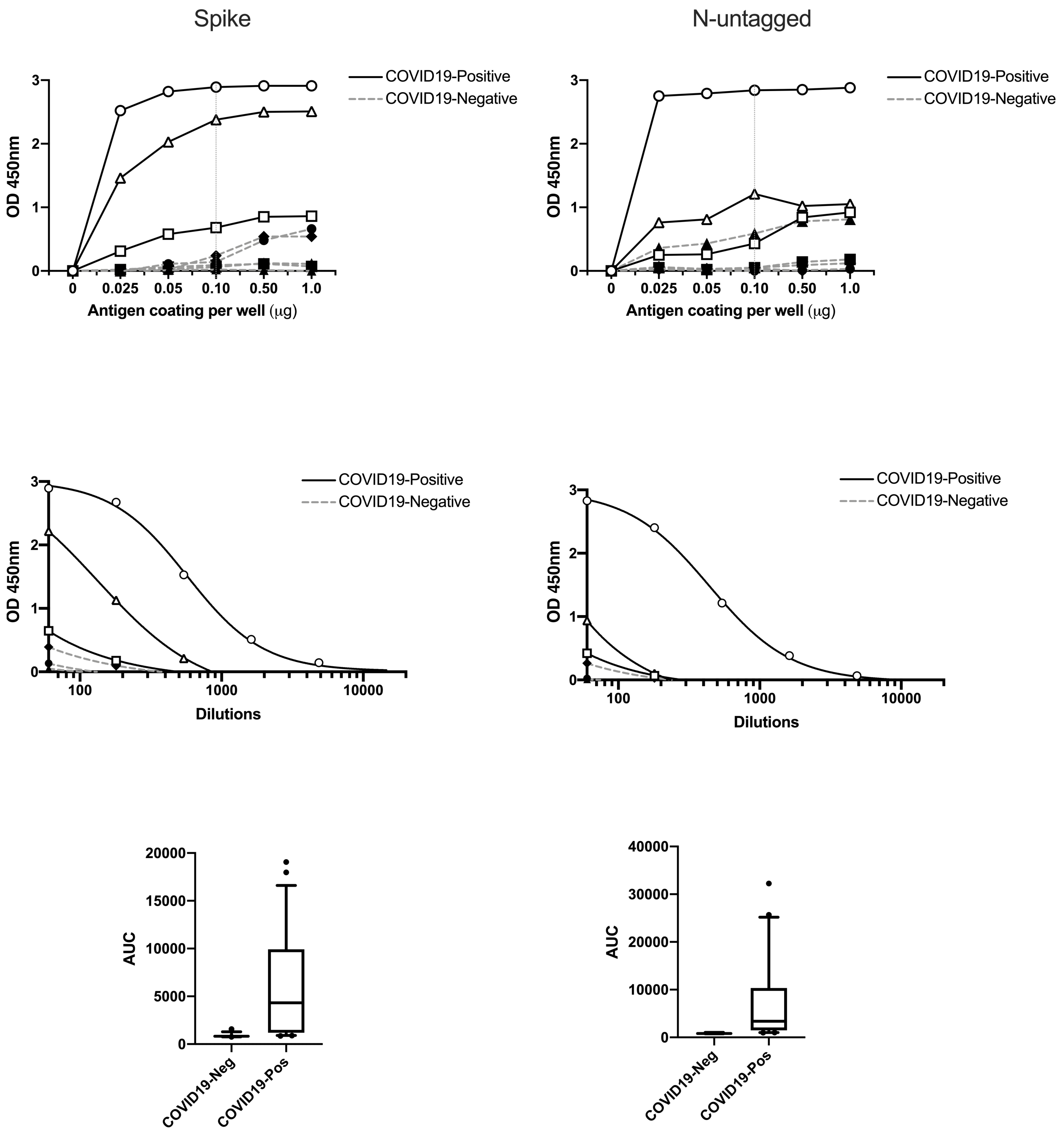

Supplementary Figure 1, related to Figure 1: Establishment of serological assay to determine positivity and endpoint titre against human SARS CoV-2. Residual stored serum samples from PCR positive and negative patient cohort were screened for reactivity against full-length spike and N-proteins. A) To determine the appropriate concentration of antigen used for plate coating, 0, 0.025, 0.05, 0.1, 0.5 and 1.0 1mg antigen per well was coated and reactivity of known seropositive and seronegative serum samples were examined. B) Subsequently, end-point titrations were performed using 0.1mg per well spike and N antigen coating. C. The area under the curve (AUC) was calculated for every sample using end point titrations against spike (n=76) and N protein (n=64), and the mean and the 95% confidence intervals are shown for all PCR positive and negative samples. OD: optical density (nanometers)

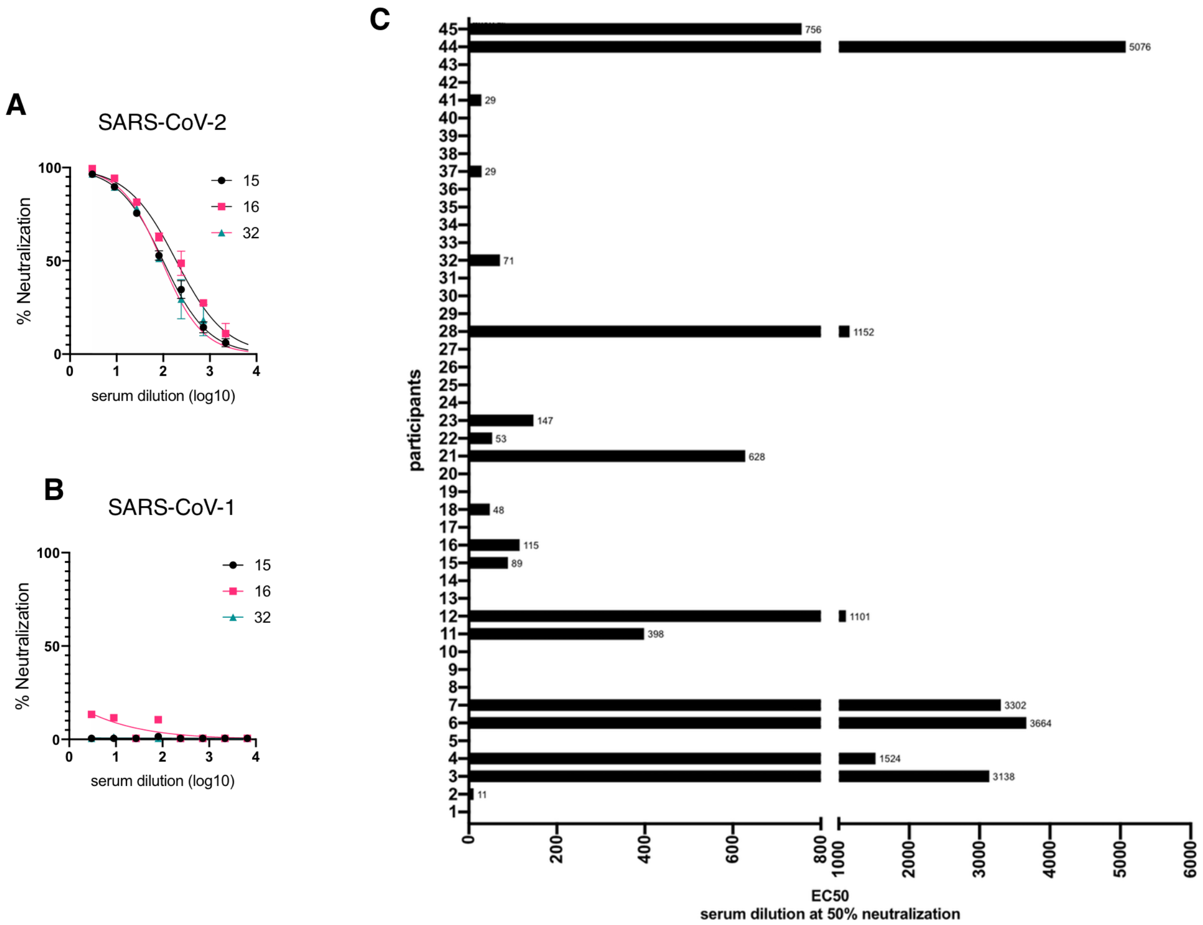

Supplementary Figure 2, related to Figure 1 : Specificity of antibody neutralizing response against SARS-CoV-2 and CoV-1.
SARS-CoV-2 **(A)** or SARS-CoV-1 **(B)** Spike protein pseudotyped viral particles were incubated with serial dilutions of heat inactivated human serum samples from Covid-19 suspected individuals (#15,16,32) in duplicates for 1h at 37˚C. 293T ACE2/TMPRSS2 expressing cells were added to each well. Following 48h incubation in a 5% CO2 environment at 37°C, the luminescence was measured using Steady-Glo Luciferase assay system (Promega). Percentage of neutralization was calculated with non-linear regression, log (inhibitor) vs. normalized response using GraphPad Prism 8 (GraphPad Software, Inc., San Diego, CA, USA). **(C)** The 50% inhibitory dilution (EC50) was defined as the serum dilution at which the relative light units (RLUs) were reduced by 50% compared with the virus control wells (virus + cells) after subtraction of the background RLUs in the control groups with cells only. The EC50 values were calculated with non-linear regression, log (inhibitor) vs. normalized response using GraphPad Prism 8 (GraphPad Software, Inc., San Diego, CA, USA).

A

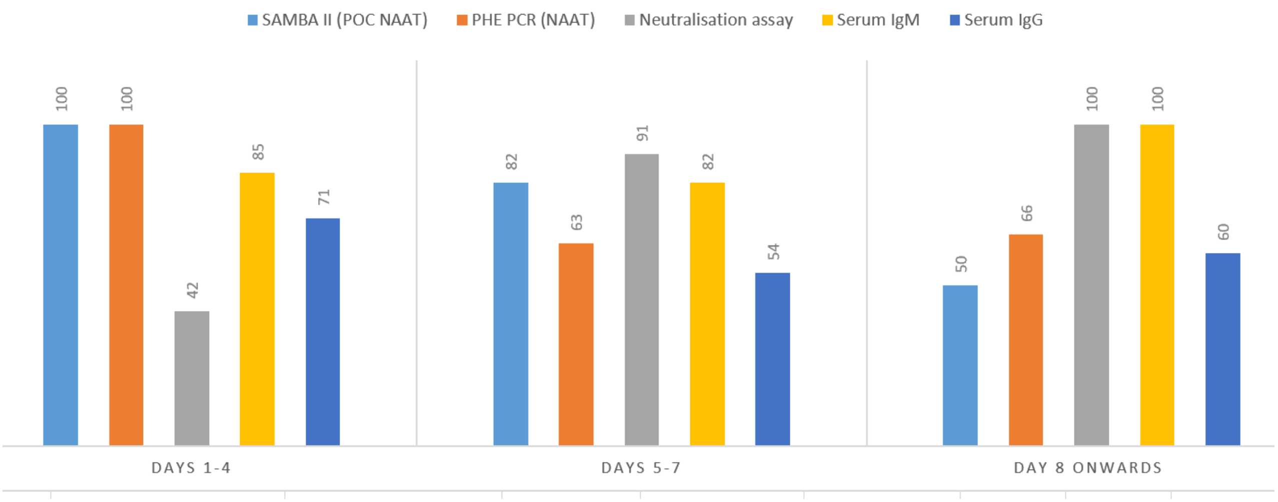

B

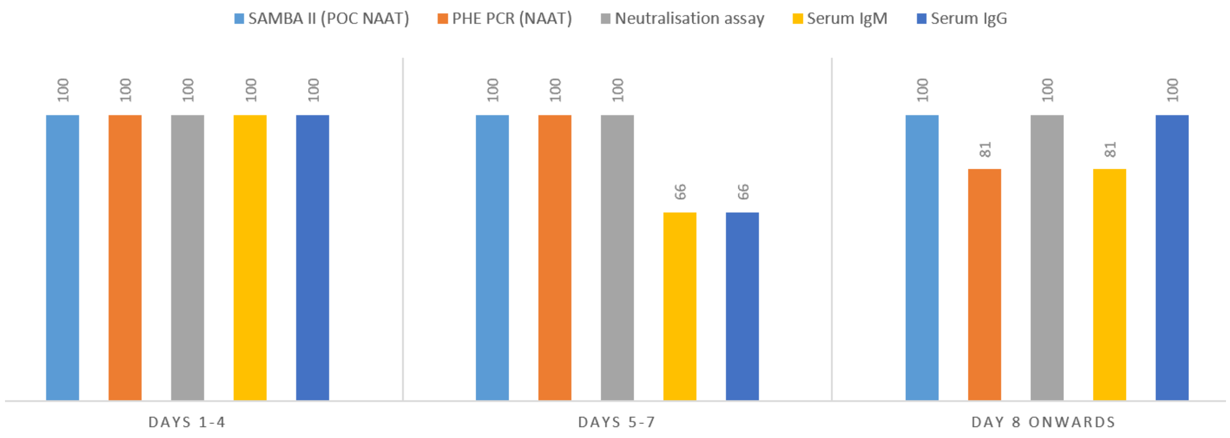

Supplementary Figure 3:, related to Figure 1. Results of assays by time since onset of symptoms (A) % of positive tests in individuals classified as COVID-19 positive by composite reference (B) % of negative tests in individuals classified as COVID-19 negative by composite reference. Here the serum IgM and IgG assay is COVIDIX SARS-CoV-2 IgM/IgG test.

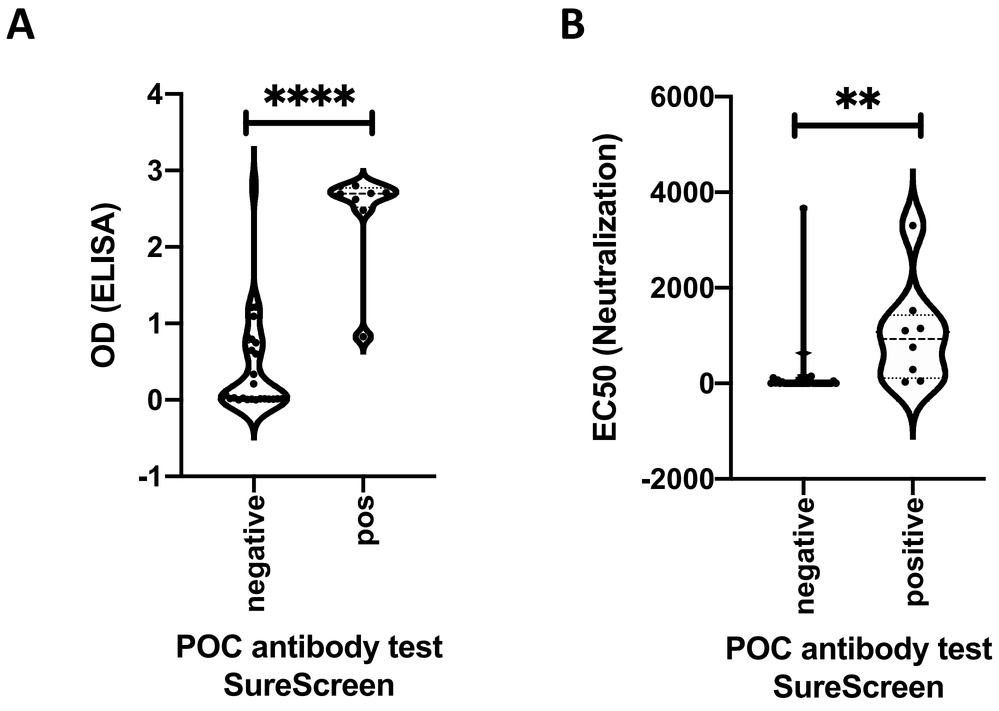

Supplementary Figure 4, related to Figure 1: Comparison of a lateral flow diagnostic test (SureScreen SARS-CoV-2 IgM/IgG test) against ELISA IgG and SARS-CoV-2 pseudotyped virus neutralisation assays on sera from patients with suspected moderate to severe COVID-19. (A) Comparison between ELISA IgG and positive/negative POC IgG band results for SureScreen SARS-CoV-2 IgM/IgG test. n=38, $p<0.0001$. (B). Comparison between EC50 dilution titre from neutralisation assay and positive/negative SureScreen SARS-CoV-2 IgM/IgG antibody band test results. n=43, $p=0.005$.

Supplementary Table 1, relates to Figure 1: Pre 2020 sera testing: ELISA optical density values for full length SARS-CoV-2 Spike (FL), Spike receptor binding domain (RBD), nucleocapsid (N), and result on testing with COVIDIX SARS-CoV-2 IgM/IgG test. Positive (from confirmed positive) and negative (pooled human sera from pre 2020) control values are given.

Sample no	FL	RBD	N	COVIDIX IgM/IgG result
1	0.95735	0.20455	0.5343	Negative
2	0.1217	0.1008	0.0746	Negative
3	0.2680	0.1300	0.1285	Negative
4	0.2511	0.0837	0.07445	Negative
5	0.10625	0.06625	0.4722	Negative
6	0.1561	0.08655	0.0927	Negative
7	1.12375	0.05785	0.40535	Negative
8	0.1432	0.0888	0.5842	Negative
9	0.49075	0.06505	0.32445	Negative
10	0.16075	0.03625	0.13485	Negative
11	0.08205	0.0504	0.07485	Negative
12	0.1956	0.23025	0.1748	Negative
13	0.1482	0.07115	0.05645	Negative
14	0.16075	0.078	1.00845	Negative
15	0.18015	0.09845	0.7598	Negative
16	0.26335	0.0693	0.38865	Negative
17	0.1864	0.18905	0.35065	Negative
18	0.1265	0.3684	0.18025	Negative
19	0.08425	0.06555	0.1378	Negative
Negative	0.297	0.054	0.387	
Positive	2.704	2.150	2.337	

Supplementary table 2, relates to Figure 3: Characteristics of 16 COVID-19 positive participants with available SARS-CoV-2 sequence data differentiated by D614G Spike mutation.

Amino acid at 614	Days post symptom onset	NAAT result	Serum Neutralisation assay	Spike ELISA	COVIDIX IgM/IgG	Sure Screen
D	7	Positive	Negative	Positive	Positive	Positive
D	7	Positive	Negative	Negative	Negative	Negative
G	1	Positive	Negative	Positive	Positive	Negative
G	11	Positive	Positive	Positive	Positive	Positive
G	7	Positive	Positive	Positive	Positive	Positive
G	1	Positive	Negative	Negative	Positive	Negative
G	9	Positive	Positive	Positive	Positive	Positive
G	21	Positive	Positive	Positive	Positive	Positive
G	1	Positive	Positive	Positive	Positive	Positive
G	1	Positive	Positive	Positive	Positive	Positive
G	7	Positive	Positive	Positive	Positive	Positive
G	7	Positive	Positive	Positive	Positive	Positive
G	1	Positive	Negative	Negative	Positive	Negative
G	1	Positive	Negative	Positive	Positive	Negative
G	1	Positive	Positive	Positive	Positive	Positive
G	6	Positive	Positive	Positive	Positive	Positive
