# Supplementary material for: Combined Point-of-Care Nucleic Acid and Antibody Testing for SARS-CoV-2 following Emergence of D614G Spike Variant
Source: Cell Rep Med. 2020 Sep 1;1(6):100099. doi: 10.1016/j.xcrm.2020.100099 (PMC7462534; doi:10.1016/j.xcrm.2020.100099)
Supplement: Document S2. Article plus Supplemental Information [file mmc2.pdf]

Cell Reports Medicine

Report

Combined Point-of-Care Nucleic Acid and Antibody Testing for SARS-CoV-2 following Emergence of D614G Spike Variant

Graphical Abstract

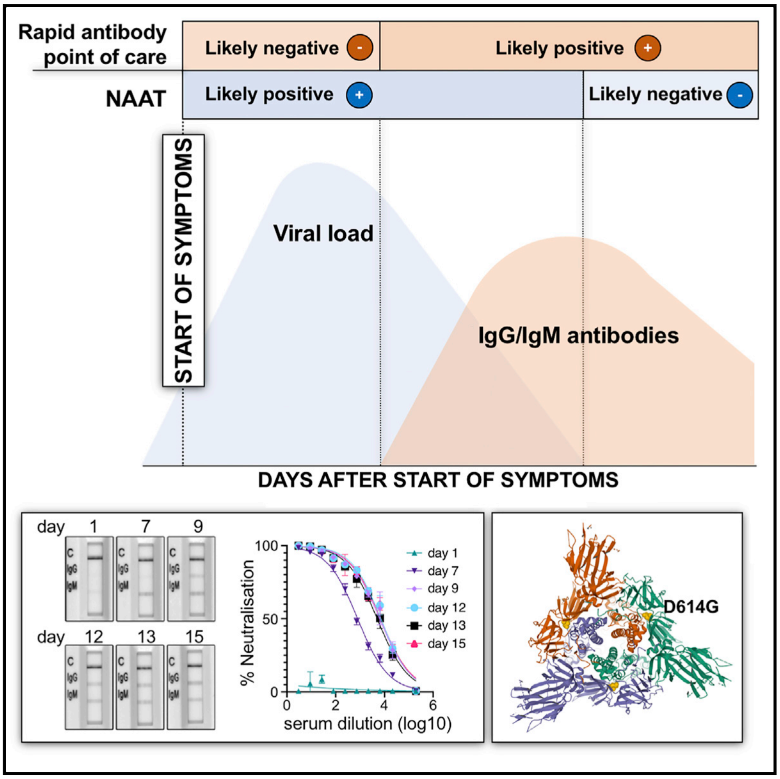

Highlights

- Combined rapid antibody + nucleic acid detection correctly diagnoses SARS-CoV-2
- Rapid antibody tests detect immune responses against SARS-CoV-2 bearing D614G
- Rapid SARS-CoV-2 antibody tests do not cross-react with antibodies to seasonal CoV
- False positivity in SARS-CoV-2 finger prick blood antibody tests can be very low

Authors

Petra Mlcochova, Dami Collier, Allyson Ritchie, ..., Ian Goodfellow, Ravindra K. Gupta, The Cambridge Institute of Therapeutic Immunology and Infectious Disease-National Institute of Health Research (CITIID-NIHR) COVID BioResource Collaboration

Correspondence

rkg20@cam.ac.uk

In Brief

Mlcochova et al. report that combined rapid nucleic acid amplification testing (NAAT) and finger prick blood antibody tests can substantially improve the diagnosis of COVID-19 as compared to NAAT alone and is able to detect the SARS-CoV-2 Spike D614G variant that dominates the pandemic.

Mlcochova et al., 2020, Cell Reports Medicine *1*, 100099
September 22, 2020 © 2020 The Author(s).
https://doi.org/10.1016/j.xcrm.2020.100099

Cell Reports Medicine

CellPress
OPEN ACCESS

Report

Combined Point-of-Care Nucleic Acid and Antibody Testing for SARS-CoV-2 following Emergence of D614G Spike Variant

Petra Mlcochova,[1,2,16] Dami Collier,[1,2,3,16] Allyson Ritchie,[4] Sonny M. Assennato,[4] Myra Hosmillo,[5] Neha Goel,[4] Bo Meng,[1,2] Krishna Chatterjee,[6] Vivien Mendoza,[6] Nigel Temperton,[7] Leo Kiss,[8] Leo C. James,[8] Katarzyna A. Ciazynska,[8] Xiaoli Xiong,[8] John A.G. Briggs,[8] James A. Nathan,[1,2] Federica Mescia,[1,2] Laura Bergamaschi,[1,2] Hongyi Zhang,[9] Petros Barmpounakis,[10] Nikos Demeris,[10,11] Richard Skells,[6,11] Paul A. Lyons,[1,2] John Bradley,[3,12] Steven Baker,[1,2] Jean Pierre Allain,[4] Kenneth G.C. Smith,[1,2] Rachel Bousfield,[13] Michael Wilson,[13] Dominic Sparkes,[13] Glenn Amoroso,[14] Effrosyni Gkrania-Klotsas,[13] Susie Hardwick,[14] Adrian Boyle,[14] Ian Goodfellow,[5] Ravindra K. Gupta,[1,2,13,15,17,18,*] and The Cambridge Institute of Therapeutic Immunology and Infectious Disease-National Institute of Health Research (CITIID-NIHR) COVID BioResource Collaboration
[1]Cambridge Institute of Therapeutic Immunology & Infectious Disease (CITIID), Cambridge, UK
[2]Department of Medicine, University of Cambridge, Cambridge, UK
[3]Division of Infection and Immunity, University College London, London WC1E 6BT, UK
[4]Diagnostics for the Real World EU, Chesterford Research Park, UK
[5]Department of Pathology, University of Cambridge, Cambridge, UK
[6]NIHR Cambridge Clinical Research Facility, Cambridge, UK
[7]Viral Pseudotype Unit, Medway School of Pharmacy, University of Kent, Kent, UK
[8]Medical Research Council Laboratory of Molecular Biology, Cambridge, UK
[9]Clinical Microbiology & Public Health Laboratory, Cambridge University NHS Hospitals Foundation Trust, Cambridge, UK
[10]Department of Statistics, Athens University of Economics and Business, Athens, Greece
[11]Cambridge Clinical Trials Unit-Cancer Theme, University of Cambridge, Cambridge, UK
[12]National Institutes for Health Research Cambridge Biomedical Research Centre, Cambridge, UK
[13]Department of Infectious Diseases, Cambridge University NHS Hospitals Foundation Trust, Cambridge, UK
[14]Department of Emergency Medicine, Cambridge University NHS Hospitals Foundation Trust, Cambridge, UK
[15]Africa Health Research Institute, Durban, South Africa
[16]These authors contributed equally
[17]Twitter: @GuptaR_lab
[18]Lead Contact
*Correspondence: rkg20@cam.ac.uk
https://doi.org/10.1016/j.xcrm.2020.100099

SUMMARY

Rapid COVID-19 diagnosis in the hospital is essential, although this is complicated by 30%–50% of nose/throat swabs being negative by SARS-CoV-2 nucleic acid amplification testing (NAAT). Furthermore, the D614G spike mutant dominates the pandemic and it is unclear how serological tests designed to detect anti-spike antibodies perform against this variant. We assess the diagnostic accuracy of combined rapid antibody point of care (POC) and nucleic acid assays for suspected COVID-19 disease due to either wild-type or the D614G spike mutant SARS-CoV-2. The overall detection rate for COVID-19 is 79.2% (95% CI 57.8–92.9) by rapid NAAT alone. The combined point of care antibody test and rapid NAAT is not affected by D614G and results in very high sensitivity for COVID-19 diagnosis with very high specificity.

INTRODUCTION

As of August 2, 2020, >18.0 million people have been infected with severe acute respiratory syndrome-coronavirus-2 (SARS-CoV-2), with >690,000 deaths.[1] The unprecedented numbers requiring SARS-CoV-2 testing has strained healthcare systems globally. There is no gold standard for the diagnosis of coronavirus disease 2019 (COVID-19). The detection of SARS-CoV-2 by nucleic acid amplification testing (NAAT) is largely done by real-time RT-PCR on nose/throat swabs in centralized laboratories. RT-PCR specimens are often batch analyzed, and the turn-around time for this test can be as long as 2–4 days in real-world settings.[2] NAAT tests from a single nose/throat swab are negative in up to 50% of patients who have computed tomography (CT) changes consistent with COVID-19 and/or positive antibodies to SARS-CoV-2.[3–5] The lack of detectable virus in upper airway samples is not only a serious barrier to making timely and safe decisions in the emergency department but it also leads to multiple swab samples being sent, frequently from the same anatomical site, resulting in additional strain on virology

This is an open access article under the CC BY license (http://creativecommons.org/licenses/by/4.0/).

laboratories. Nonetheless, NAAT remains important in identifying infectious individuals. In addition, in severely ill patients, tracheobronchial samples may be NAAT$^+$, even when the nose/throat swab is negative.[4,6]

Multiple factors may contribute to negative results by NAAT, including test sensitivity, sampling technique, and timing of the sampling in the disease course.[6] The viral load in the upper respiratory tract is detectable from ~4 days before symptoms[7] and frequently wanes after 1 week post-symptom onset.[8,9] Similarly, a case series from Germany found the detection rate by RT-PCR was <50% after 5 days since onset of illness.[10] A proportion of patients develop secondary deterioration in clinical condition, requiring hospitalization and respiratory support, at a time when immune pathology rather than direct pathology related to viral replication is thought to be dominant.[9,11]

An antibody response to SARS-CoV-2 is detectable 6 days from infection and is almost always neutralizing.[12,13] Antibody-based diagnosis of COVID-19 shows increasing sensitivity in the latter part of the infection course, when NAAT on nose/throat samples is more likely to be negative.[14–17] As a result, the diagnosis of infection and the identification of infectivity would benefit from a combination of virologic and immunologic markers to inform patient initial triage and subsequent management. It is critical to determine whether a rapid point of care combined antibody and nucleic acid testing strategy could improve diagnosis.

We previously evaluated the diagnostic accuracy of the SAMBA (simple amplification-based assay) II SARS-CoV-2 rapid test compared with the standard laboratory RT-PCR and found similar accuracy, with a turnaround time of 2–3 h, even in real-world settings.[18] Several studies have reported head-to-head comparisons of immunochromatographic lateral flow immunoassays (LFAs).[15–17,19] These assays are inexpensive to manufacture and provide a binary positive/negative result, thereby lending themselves well to point-of-care (POC) testing. Even though they have variable performance and in general are negative in the early phase of infection, they become highly sensitive in the later stage of illness,[15–17,19] and some are also highly specific.

In this study, we evaluated the diagnostic performance of a POC combination comprising NAAT and antibody testing against a composite reference standard of laboratory RT-PCR and a serum neutralization assay. Notably, SARS-CoV-2 viruses with a D-to-G mutation in Spike at position 614 have increased in prevalence globally.[20] Cryoelectron microscopy (cryo-EM) studies suggest that D614 may play a role in Spike intermolecular stability,[21] potentially contributing to increased infectivity.[20] Given that POC antibody tests were designed to detect antibodies to the wild-type S protein, we also aimed to investigate whether SARS-CoV-2 infections with D614G Spike mutant virus could be diagnosed by POC antibody tests.

RESULTS

In phase one, 45 prospectively recruited participants in the COVIDx study with suspected COVID-19 disease had nose/throat swabs specimens tested for nucleic acid and stored sera for antibody testing. Samples at hospital admission were collected at a median of 7 (interquartile range [IQR] 7–13) days after illness onset. The sera from 42.2% (19/45) participants showed neutralizing antibody response against SARS-CoV-2 Spike protein pseudotyped virus infection in a neutralization assay using a cut-off of 50% inhibition at 1:4 dilution (Figure 1A). The sera of 26 participants showed no neutralizing response (Figure 1B). The neutralization ability of participants' sera was compared with an in-house ELISA immunoglobulin G (IgG) assay for Spike-specific antibodies based on a recently reported method[22] (Figure S1), and significant association between positive results in both assays was demonstrated (Figure 1C, $p < 0.0001$). Figures 1D–1G show significant associations between the POC antibody test result and both ELISA ($p < 0.0001$) and neutralization ($p < 0.0025$) assays. POC antibody testing showed no cross-reactivity in sera obtained before the pandemic (Table S1). The neutralization assay also demonstrated a lack of cross-reactivity with SARS-CoV-1 on a limited subset of sera (Figure S2).

Results from the 4 IgG antibody assays used in this study were confirmed (4 or 3 concordant) in 38/45 samples, and, against this classification, neutralization (Figures 1A–1C), spike ELISA[22] (Figures 1C, 1D, 1F, and S1), Surescreen, and COVIDIX Healthcare assays gave a correct result in 100%, 97.4%, 92.1%, and 86.8% of cases, respectively, justifying the choice of the neutralization assay as part of a composite reference standard.

A total of 53.3% (24/45) of participants had COVID-19 disease, as determined by the composite reference standard (lab RT-PCR and neutralization assay). The median age of the patients was 73.5 (IQR 54.0–86.5) years in those with SARS-CoV-2 infection by our composite reference standard and 63.0 (IQR 41.0–72.0) years in those without disease (Table 1). C-reactive protein (CRP) and procalcitonin were significantly higher in confirmed COVID-19 patients, and classical chest radiograph appearances were more common in confirmed COVID-19 patients (Table 1, $p < 0.001$). However, 6/24 (25%) had normal or indeterminate chest radiographs in the confirmed COVID-19 group.

As expected from the clinical study inclusion criteria, >80% of patients presented with influenza-like illness (ILI) with documented fever and ~33% had clinical or radiological evidence of pneumonia (Table 1). Highly experienced internal medicine physicians were caring for suspected COVID-19 cases at our institution, and this was partly due to the significant comorbidities in the local population that mandated a broad differential diagnostic approach in hospitalized individuals (Table 1). Among the patients with COVID-19, one suffered from rheumatoid arthritis and was immunosuppressed with prednisolone. Among the patients without COVID-19, 5 were immunosuppressed for the following conditions: psoriatic arthritis (usekinumab, anti-interleukin-12 [IL-12], IL-23), multiple myeloma (lenalidomide and dexamethasone), lymphoma (cyclosporin), hypersensitivity pneumonitis (mycofenalate and prednisolone), and renal transplant (mycofenalate and tacrolimus). No patients in the study were under treatment with the anti-B cell monoclonal antibody rituximab.

During the peak of the first wave, routine respiratory virus testing was halted at our institution due to the demands of SARS-CoV-2 testing and low seasonal prevalence of these pathogens. Multiplex PCR for other respiratory viral pathogens was performed in only 8 participants. Seven of these

CellPress
OPEN ACCESS

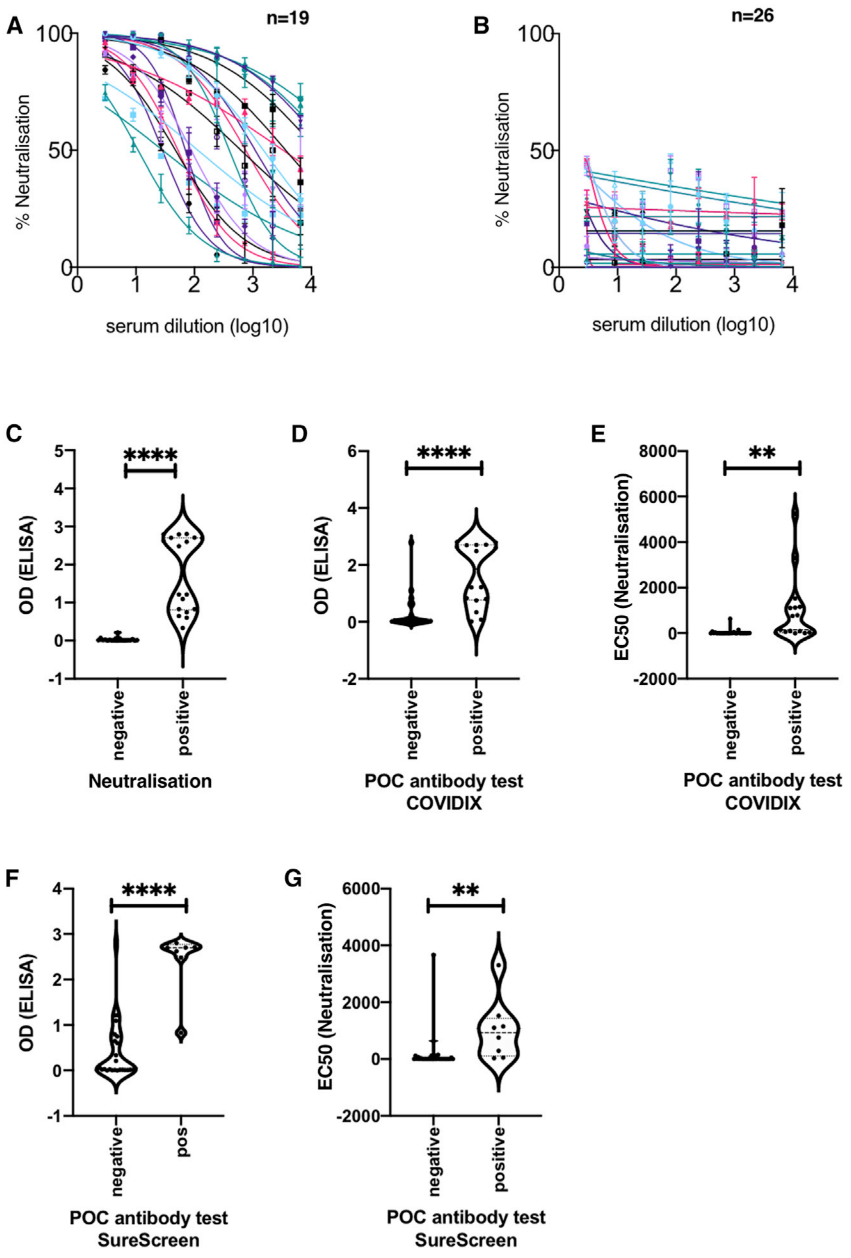

Figure 1. Antibody Detection for SARS-CoV-2: Cross-Validation of Lateral Flow Diagnostic Tests (POC Antibody Tests) with ELISA and SARS-CoV-2 Pseudotype Virus Neutralization Assays

(A and B) Serum from COVID-19 suspected participants inhibited (n = 19) (A) or did not inhibit (n = 26) (B) SARS-CoV-2 pseudotype virus infection in a neutralization assay. Serum from a healthy donor was used and a negative control. The error bars represent SEMs.

(C) Comparison between ELISA and positive/negative results from neutralization assay; n = 37, $p < 0.0001$.

(D) Comparison between ELISA Spike protein reactivity and positive/negative POC antibody test results (COVIDIX SARS-CoV-2 IgM/IgG test); n = 38, $p < 0.0001$.

(E) Comparison between half-maximal effective concentration (EC_{50}) dilution titer from neutralizing assay and positive/negative POC antibody test results (COVIDIX SARS-CoV-2 IgM/IgG test); n = 44, p = 0.0025.

(F) Comparison between ELISA IgG and positive/negative POC IgG band results for SureScreen SARS-CoV-2 IgM/IgG test; n = 38, $p < 0.0001$.

(G) Comparison between EC_{50} dilution titer from neutralization assay and positive/negative SureScreen SARS-CoV-2 IgM/IgG antibody band test results; n = 43, p = 0.005. The assays were performed in duplicate.

participants were negative and one participant tested positive for influenza A.

The overall COVID-19 diagnosis rate (positive predictive agreement) by rapid nucleic acid testing was 79.2% (95% confidence interval [CI] 57.8–92.9), decreasing from 100% (95% CI 59.0%–100%) for days 1–4 to 50.0% (95% CI 11.8–88.2) for days 9–28 post-symptom onset (Table 2; Figure S3). When IgG/IgM rapid tests were combined with NAAT, the overall positive predictive agreement increased to 100% (95% CI 85.8–100) (Table 2). Additional cases of COVID-19 detected in NAAT^{-} patients were identified by POC tests under investigation (Figure 2). Among 21 COVID-19^{-} individuals, there were 3 false positive results for 1 POC antibody test and 1 false positive result for the other, resulting in positive predictive values of 88.9% and 96.0%, respectively, for the 2 POC antibody/SAMBA II NAAT combinations.

Those with positive NAAT and sequence available were predominantly infected with strains containing the D614G mutation in Spike, downstream of the receptor-binding domain and located on the Spike surface (Figures 3A and 3B). A total of 14/24 (58.3%) patients deemed to be COVID-19^{+} by the reference composite standard were positive by both rapid NAAT and antibody testing, and 14/14 were infected with strains bearing D614G, indicating that POC serological tests were able to detect infections with this variant.

To understand the relationship between POC band intensity and neutralization activity further, we identified 3 participants (all infected with D614G Spike mutant) with stored samples at multiple time points in their illness (Figure 4). Two individuals were sampled from early after symptom onset, and the third presented 3 weeks into illness. In the first two cases (Figures 4A–4F), we observed an increase in neutralization activity over time that was mirrored by band intensities on rapid POC antibody testing. As expected, IgM bands arose early on, with IgG following closely. Of note, in patient 1, there was a weakly detectable IgM band by rapid test with no serum neutralization activity

Table 1. Characteristics of Participants in Diagnostic Accuracy Study

	COVID-19⁺	COVID-19⁻	
	n = 24	n = 21	p[a]
Male sex (%)	14 (58.3)	9 (42.9)	0.30[b]
Median age, y (IQR)	73.5 (54.0–86.5)	63.0 (41.0–72.0)	0.03
Influenza-like illness with documented fever	20 (83.3)	17 (81.0)	0.84
Clinical or radiological evidence of pneumonia	10 (41.7)	7 (33.3)	0.57
Immunosuppressed			0.053
Yes	1 (4.2)	5 (23.8)	–
No	23 (95.8)	16 (76.2)	–
Cardiovascular disease	6 (25.0)	2 (9.5)	0.25
Chronic respiratory disease	5 (20.8)	6 (28.6)	0.73
Chronic renal disease	4 (16.7)	2 (9.5)	0.67
Diabetes mellitus	6 (25.0)	3 (14.3)	0.47
Median SpO_2, % (IQR)	95.0 (92.5–96.0)	96.0 (94.0–98.0)	0.09
Median FiO_2, % (IQR)	0.21 (0.21–0.24)	0.21 (0.21–0.21)	0.40
Median PaO_2, kPa (IQR)	5.0 (3.0–9.1)	7.2 (3.8–9.0)	0.30
Median PaO2: FiO_2 (IQR)	20.5 (13.3–32.9)	30.9 (18.1–36.2)	0.09
Median respiratory rate, breaths/min (IQR)	22.0 (19.0–27.5)	20.0 (17.0–23.0)	0.06
Median heart rate, beats/min (IQR)	86.0 (77.5–99.5)	88.0 (78.0–107.0)	0.44
Median systolic BP, mmHg (IQR)	139.5 (117.5–149.0)	135.0 (119.0–152.0)	0.90
Median duration of illness, days (IQR)	7 (1–8)	10 (3–14)	0.10
Median Hb, g/dL (IQR)	12.9 (12.0–13.8)	13.1 (11.6–14.1)	0.46
Median WCC, × 10^9/L (IQR)	7.0 (5.0–8.0)	9.0 (7.0–14.0)	0.08
Median lymphocyte count, ×10^9/L (IQR)	0.8 (0.5–1.2)	1.2 (0.8–1.5)	0.12
Median platelet count, ×10^9/L (IQR)	213.5 (188.5–303.5)	271.0 (186.0–305.0)	0.59
Median ferritin, μg/L (IQR)	684.7 (206.2–1059.1)	112.3 (49.6–323.6)	0.02
Median CRP, mg/L (IQR)	72.0 (28.5–214.5)	12 (4.0–53.0)	0.004
Median procalcitonin, ng/mL (IQR)	0.2 (0.1–0.6)	0.0 (0.0–0.1)	0.03
Radiological findings			<0.001[b]
Normal	2 (8.3)	9 (42.9)	–
Indeterminate	4 (16.7)	3 (14.3)	–
Classic	18 (75.0)	3 (14.3)	–
Non-COVID	0 (0.0)	6 (28.5)	–

COVID-19 status is based on composite reference standard test of nose/throat swab SARS-CoV-2 RT-PCR + serum neutralization of pseudovirus-bearing SARS-CoV-2 Spike. BP, blood pressure; Hb, hemoglobin; WCC, white blood cell count.
[a]Wilcoxon rank-sum test used except where indicated.
[b]Chi-square test.

(Figures 4A and 4B). Over time, the band intensity for IgM and IgG increased along with the serum neutralization activity. In the individual presenting 21 days into illness (Figures 4G–4I), only IgG was detected with rapid POC antibody testing, and, as expected, the band intensity did not increase over the following days.

In phase 2, we performed a prospective evaluation of combined testing in 128 patients presenting with possible COVID-19 from July 13 to 27, 2020. Their clinical presentation was less severe and diagnoses broader than in phase 1 (Table 3), with cardiovascular and gastrointestinal disease significantly represented and respiratory disease representing just 60% of cases—likely as a result of the increased appreciation of diverse presentations of COVID-19 disease.[23] Patients did have significant comorbidities and ~10% were immunosuppressed, although without B cell-depleting agents (Table 3). By this time, the POC NAAT test had been validated in a head-to-head study against the lab RT-PCR and entered routine use,[18] replacing the RT-PCR. Given the need to further assess the specificity of the POC antibody tests in routine clinical practice and with fresh blood rather than serum, we compared the performance of POC antibody tests on finger prick blood against serum neutralization (Figures 5A and 5B).

In this second phase, there was only one NAAT positive patient, who was also positive by both POC antibody tests and serum neutralization. There were three NAAT⁻ individuals presenting with respiratory symptoms who had positive POC antibody tests by both COVIDIX and SureScreen, along with serum neutralization activity. The POC antibody tests showed 100% negative predictive agreement with serum neutralization and the kappa correlation between POC antibody tests and serum neutralization was extremely high, at 0.97.

DISCUSSION

Here, we have shown that POC NAAT testing in combination with antibody detection can significantly improve the diagnosis of COVID-19. The overall positive predictive agreement against the composite reference standard under clinical trial conditions was ~79% for rapid NAAT testing of nose/throat swab samples, reaching 100% with a combined approach of rapid NAAT testing

Table 2. Individual and Combined Diagnostic Accuracy of POC Rapid NAAT-Based and Antibody Tests According to Time from Initial Symptoms

	% (95% CI)			
	Days 1–4	Days 5–8	Days 9–28	Overall
	n = 14	n = 14	n = 17	N = 45[a]
SAMBA II NAAT				
Positive predictive agreement	100 (59.0–100)	81.8 (48.2–97.8)	50.0 (11.8–88.2)	79.2 (57.8–92.9)
Negative predictive agreement	100 (59.0–100)	100 (29.2–100)	100 (71.5–100))	100 (83.9–100)
COVIDIX IgM and IgG				
Positive predictive agreement	100 (59.0–100)	90.9 (58.7–99.8)	100 (54.1–100)	95.8 (78.9–99.9)
Negative predictive agreement	100 (59.0–100)	66.7 (9.4–99.2)	81.8 (48.2–97.7)	85.7 (63.7–97.0)
SAMBA II NAAT and COVIDIX IgM and IgG				
Positive predictive agreement	100 (59.0–100)	100 (71.5–100)	100 (54.1–100)	100 (85.8–100)
Negative predictive agreement	100 (59.0–100)	66.7 (9.4–99.2)	81.8 (48.2–97.7)	85.7 (63.7–97.0)
SureScreen IgM and IgG[a]				
Positive predictive agreement	42.9 (9.9–81.6)	90.9 (58.7–99.8)	100 (54.1–100)	79.2 (57.8–92.9)
Negative predictive agreement	100 (54.1–100)	66.7 (9.4–99.2)	100 (69.2–100)	94.7 (74.0–99.9)
SAMBA II NAAT and SureScreen IgM and IgG[a]				
Positive predictive agreement	100 (59.0–100)	100 (71.5–100)	100 (54.1–100)	100 (85.8–100)
Negative predictive agreement	100 (54.1–100)	66.7 (9.4–99.2)	100 (69.2–100)	94.7 (74.0–99.9)

Positivity predictive agreement is the percentage of positive test results in samples deemed positive by the composite reference standard. Negative predictive agreement is the percentage of negative test results in samples deemed negative by the composite reference standard.
[a]43 of 45 patients had SureScreen antibody results.

and either of the 2 POC lateral flow-based antibody tests. The specificity of the combined approach was 85%–95% on stored serum under clinical trial conditions and 100% on finger prick blood in routine clinical care.

As expected, nucleic acid detection in nose/throat samples was highest in those presenting within the first few days (100% in samples taken in the first 4 days after symptom onset). Conversely, antibody detection by LFA increased with time since symptom onset, with 100% efficacy beyond the 9th day post-symptoms. One study reported that combined lab-based RT-PCR with lab-based antibody testing could increase the sensitivity for COVID-19 diagnosis from 67.1% to 99.4% in hospitalized patients.[24] However, in that study, this assessment of sensitivity was made using clinical diagnosis. A major strength of the present study is the use of an objective reference standard that included NAAT and serum neutralization, a phenotypic test for the functionality of antibodies. This assay was shown to be robust and accurate, using a recently described ELISA method for SARS-CoV-2 IgG detection that is now used globally.[22]

The D614G Spike mutant has spread globally. Wild-type Spike protein antigen is used in the development and validation of POC antibody tests, including those tested here. Of critical importance is the fact that both POC antibody tests (and ELISA) were able to detect antibody responses in patients infected with the D614G Spike mutant and that the band intensity of POC testing increased with neutralization activity in these individuals. Given that POC antibody tests are far cheaper and simpler to deploy, they will likely be used in low-resource settings that do not have access to NAAT.[25] Demonstration that POC antibody LFA tests can detect the D614G spike mutant is therefore of importance.

The use of antibody tests for COVID-19 diagnosis in hospitals has been limited for a number of reasons. First, we know from SARS-CoV-1 that previous humoral immunity to HCoV OC43 and 229E can elicit a cross-reactive antibody response to N of SARS-CoV-1 in up to 14% of people tested in cross-sectional studies,[26] and previous exposure to HCoV can rarely elicit a cross-reactive antibody response to the N and S proteins of SARS-CoV-2.[16,27] Second, antibody tests do not achieve the same detection rates as nucleic acid-based tests early in infection, as humoral responses take time to develop following viral antigenic stimulation. However, by day 6 post-symptom onset, detection of IgG to Spike protein has been reported to reach 100% sensitivity,[12] and this is useful in cases with immune-mediated inflammatory disease in which RT-PCR on respiratory samples is often negative—for example, in the recently described Kawasaki-like syndrome called PIMS (pediatric inflammatory multisystem syndrome).[28]

In phase one (COVIDx trial), we tested stored sera rather than whole-blood finger prick, although this was intentional, given the caution needed in interpreting LFAs and concern regarding potential cross-reactivity of antibodies and poor specificity. Although SARS-CoV-2 ELISA testing of our pre-pandemic sera did reveal occasional N reactivity to SARS-CoV-2, likely due to cross-reactivity with seasonal CoV, these samples were negative on POC antibody testing. However, the specificity of the COVIDIX test was estimated at only 85%, compared to a more acceptable 95% for SureScreen. We therefore carried out a prospective evaluation of POC antibody testing on finger prick blood in 128 suspected cases of COVID-19 to further evaluate the specificity of both tests in routine clinical practice. We found no false positives in patients whose sera were non-neutralizing. This is consistent with an estimated specificity of >99% with the SureScreen assay observed in an independent analysis using stored pre-pandemic sera.[29] The greater incidence of false positive POC antibody tests, predominantly with COVIDIX, on stored sera as compared to fresh finger prick blood may be due to processing and storage of sera, contamination of sera

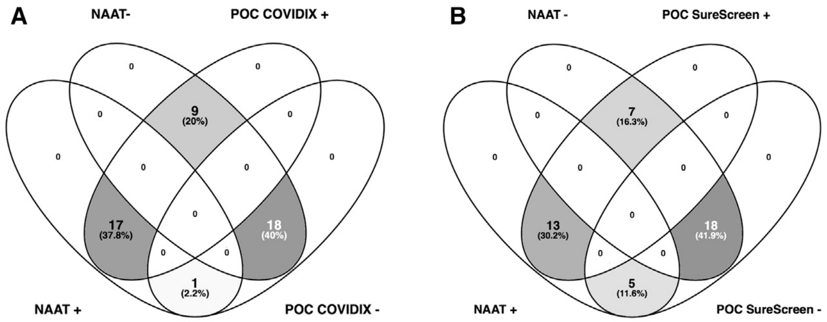

Figure 2. Venn Diagrams Comparing Positive and Negative Diagnostic Test Results in Hospitalized Patients

Testing by NAAT and POC antibody testing by (A) COVIDIX Healthcare IgM/IgG kit (n = 45) and (B) SureScreen IgM/IgG kit (n = 43).

with other blood products, or other causes, including patient factors that differed between the two phases. Nevertheless, now that we are in a low incidence period, it is advisable to perform confirmation testing using an alternative platform for either a single positive antibody or NAAT test, as is now the policy at our institution. One should note in particular that antibody tests may be negative in patients with immunosuppression, highlighting that patient factors can influence the interpretation of results and that alternative diagnoses should be considered.

We envisage a deployment approach whereby both test samples, finger prick whole blood and nose/throat swab, are taken at the same time on admission to the hospital. The finger prick antibody test result is available within 15 min. Due to the possibility of false positive results from POC serology testing, a positive POC antibody test result as the only positive marker should ideally be confirmed with a second rapid POC test/laboratory IgG/IgM test before movement to a COVID-19 area or recruitment into a clinical

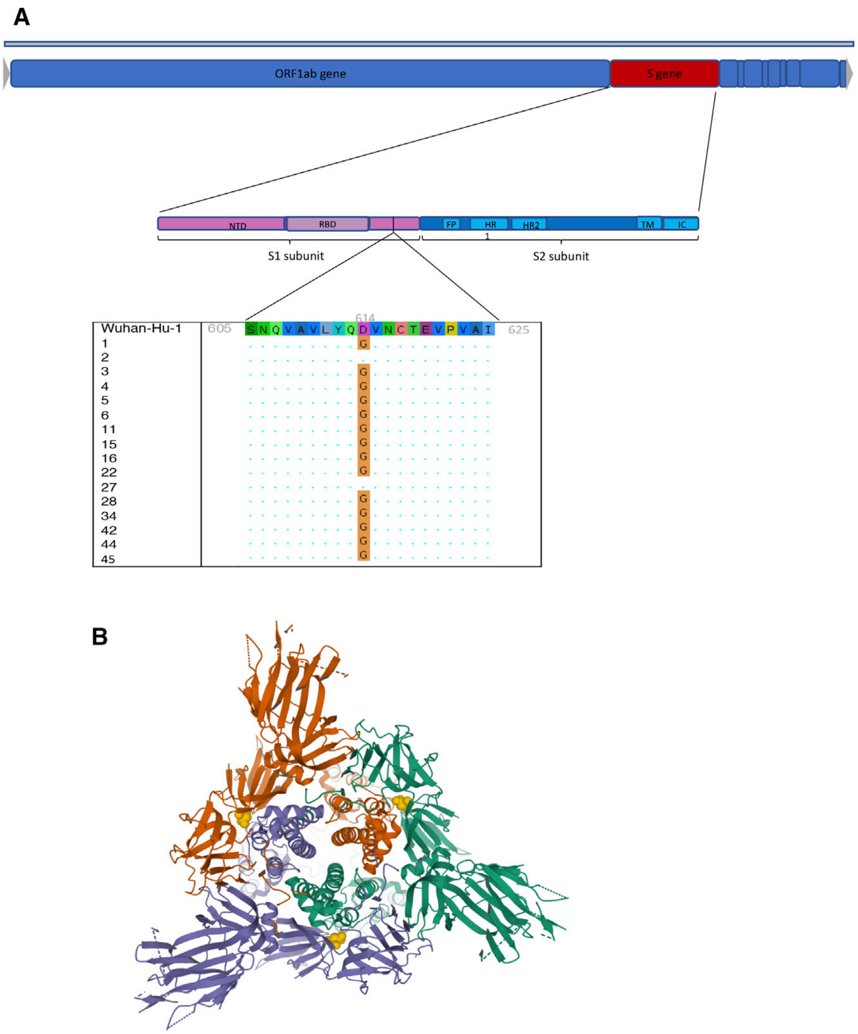

Figure 3. Spike D614G Characterization in the Phase 1 Clinical Cohort (A) Genome map of SARS-CoV-2, with overall topography of Spike expanded. FP, fusion peptide; HR1, heptad repeat 1; HR2, heptad repeat 2; IC, intracellular domain; NTD, N-terminal domain; RBD, receptor-binding domain; TM, transmembrane region. The aligned sequence of 10 amino acids on either side of D614 is shown for 16 participants for whom sequence data were available. A dot represents where the amino acid is unchanged from wild type, the mutant glycine is represented by G.

(B) Top view of SARS-CoV-2 Spike glycoprotein trimeric structure in a closed state, with position 614 in yellow in each protomer. Structure determined by cryoelectron microscopy. RCSB PDB: 6VXX.

CellPress
OPEN ACCESS

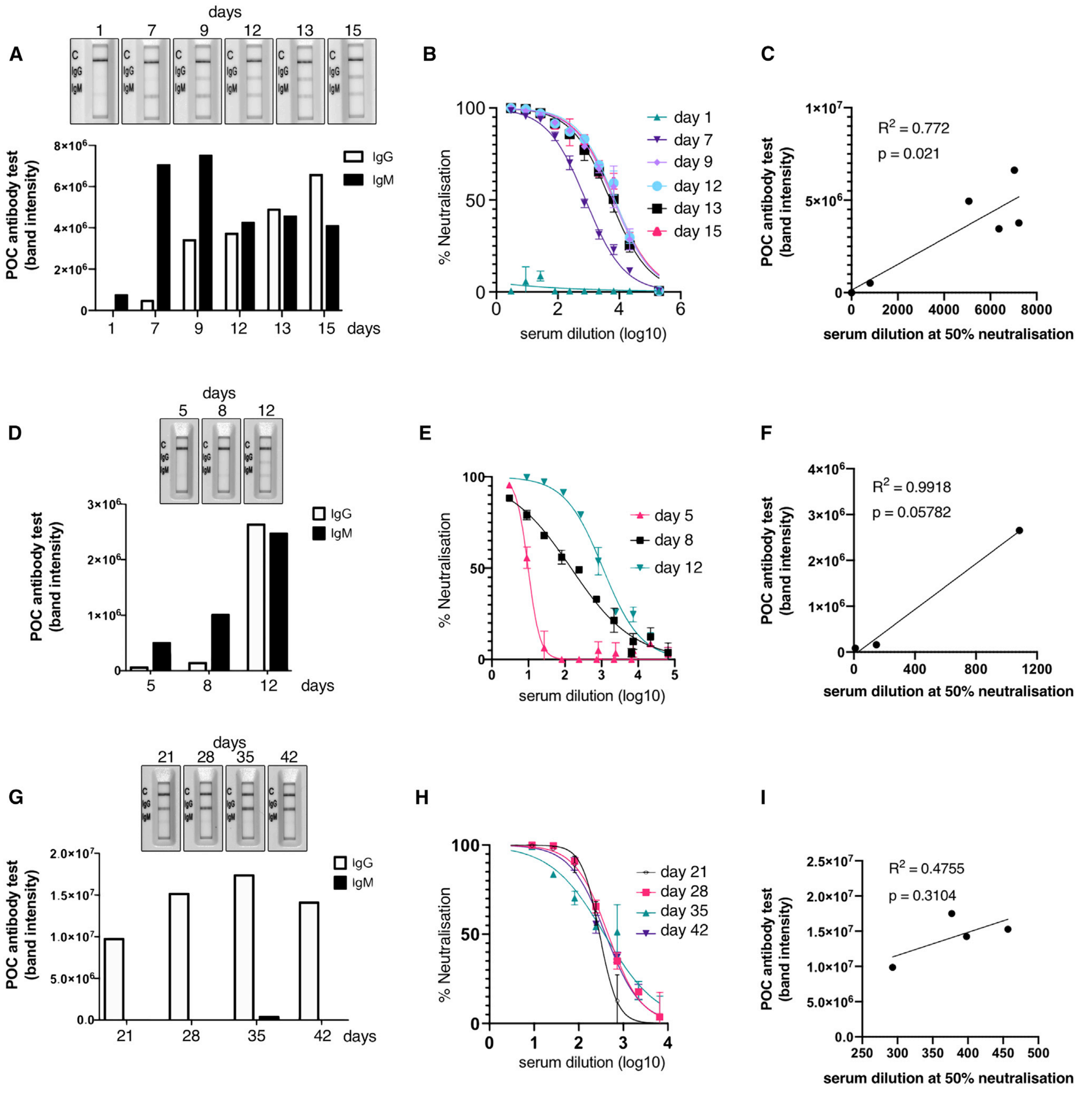

Figure 4. Longitudinal Antibody Responses in Patients Infected with D614G Mutant SARS-CoV-2 Detected by Rapid Lateral Flow and Neutralization Assays

(A, D, and G) An immunochromatographic lateral flow rapid diagnostic test (POC antibody test-COVIDIX SARS-CoV-2 IgM IgG test) on longitudinal samples in individual patients detecting SARS-CoV-2 IgM and IgG bands. Band intensities were acquired using the ChemiDoc MP Imaging System and quantified using Image Lab software.

(B, E, and H) SARS-CoV-2 pseudotyped virus neutralization assay from longitudinal serum samples in individual patient examples. The assays were performed in duplicate. The error bars represent SEMs.

(C, F, and I) Comparison of IgG band intensities from lateral flow rapid diagnostic test with EC_{50} neutralization titers from SARS-CoV-2 pseudotyped virus neutralization assay in individual patients. The correlations were estimated by linear regression analysis.

Table 3. Characteristics of 128 Individuals Hospitalized with Suspected COVID-19 during Implementation of Combined POC Testing

Characteristic	n
Male gender (%)	42.2
Median age, y (IQR)	67 (50.8–80.0)
Median SpO_2, % (IQR)	96 (95–97)
Median fiO_2 (IQR)	0.21 (0.21–0.21)
Maximal additional ventilatory support	
Nasal cannulae	24
Face mask	7
LTOT/NIV	4
Intubation	1
Median duration of illness, days (IQR)	2.5 (1–7)
$NAAT^+$ (%)	2 (1.6)
Neutralization positive (%, n = 101)	8 (7.9)
COVIDIX Healthcare IgG/M^+ (%)[a]	6 (3.9)
SureScreen IgG/M^+ (%)[a]	6 (3.1)
Median lymphocyte count, $\times 10^9$/L (IQR)	1.3 (0.76–1.76)
Median CRP, mg/L (IQR)	46 (15–129)
Comorbidities	
Cardiovascular disease	44 (34.3)
Chronic respiratory disease	62 (48.4)
Chronic kidney disease	11 (8.6)
Diabetes mellitus	24 (18.8)
Immune suppression	13 (10.2)
Diagnosis	
Respiratory	61
Cardiovascular	16
Gastrointestinal	13
Genitourinary	7
Other	30
NEWS score	2 (1–5)
Chest radiograph findings (n = 114)	
Normal	59
Indeterminate	31
Classic	0
Non-COVID-19	24
CT findings (n = 24)	
Normal	3
Indeterminate	7
Classic	1
Non-COVID-19	13

LTOT, long-term oxygen therapy; NAAT, nucleic acid amplification testing; NEWS; national early warning score; NIV, non-invasive ventilation.
[a]Testing done on stored serum due to finger prick test failure.

treatment study. At our institution, additional diagnostic data from chest imaging and blood tests such as lymphocyte count and CRP are considered in clinical decision making when assessing patinets for COVID-19. Further swabs for NAAT testing are also taken where possible.

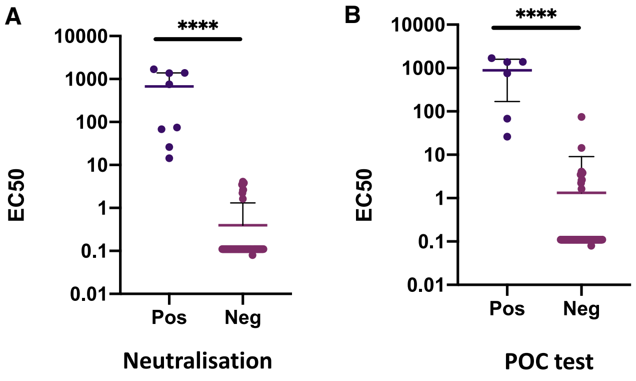

Figure 5. Distribution of Serum Neutralization Activity against SARS-CoV-2 in Hospitalized Patients during the Implementation Phase

(A) Neutralization EC_{50} dilution titer interpreted as positive or negative using a cutoff for positive neutralization of 1:4 dilution.
(B) Neutralization data for individual patients stratified by POC antibody test result (both tests were fully concordant in phase 2). The data points represent the reciprocal dilution of serum required to inhibit 50% of infection by lentivirus pseudotyped with the SARS-CoV-2 Spike glycoprotein. The assays were performed in duplicate. The line represents the mean and the bar represents the standard deviation (n = 101 sera tested).

A confirmed positive NAAT result remains critical not only to identify early infection but, more important, to triage infectious patients to be isolated from other patients and be handled with particular care by staff. NAAT is also valuable in milder and asymptomatic cases, given that severity appears to correlate with the magnitude of antibody responses.[16,30]

In conclusion, rapid combined testing could be important in the diagnosis and management of COVID-19, particularly given that the pandemic is not well controlled in many parts of the world and as diverse manifestations of disease emerge.

Limitations of Study

This study was limited by the fact that it was conducted at a single center with relatively small numbers of individuals in the clinical study (phase 1), largely due to a lack of available stored serum. Phase 1 of the study used stored serum, in which there was a higher false positive rate than phase 2, in which whole blood was used. The implementation study (phase 2) had greater numbers and was able to effectively demonstrate the high specificity and the very low false positive rate of the POC antibody tests on whole blood. However, it was hampered by the low incidence of COVID-19 infection during the period it was undertaken. This limited the further evaluation of the sensitivity of the combined approach. There was also a lack of data on repeated sampling and sampling from deeper respiratory sites in those suspected cases who were $NAAT^-$. Future larger studies are warranted.

STAR★METHODS

Detailed methods are provided in the online version of this paper and include the following:

- KEY RESOURCES TABLE
- RESOURCE AVAILABILITY
  - Lead Contact
  - Materials Availability
  - Data and Code Availability
- EXPERIMENTAL MODEL AND SUBJECT DETAILS
  - Clinical Study
  - Cell lines
  - Ethical approval
- METHOD DETAILS
  - Test methods
  - Next generation sequencing of SARS-CoV-2 isolates in nose/throat swabs
- QUANTIFICATION AND STATISTICAL ANALYSIS
  - Enzyme-linked immunosorbent assay (ELISA) quantification
  - COVIDIX 2019 nCoV IgG/IgM Test band density
  - Quantification of neutralisation sensitivity
  - Assessment of neutralisation assay performance
  - Analyses
- ADDITIONAL RESOURCES

SUPPLEMENTAL INFORMATION

Supplemental Information can be found online at https://doi.org/10.1016/j.xcrm.2020.100099.

ACKNOWLEDGMENTS

We would like to thank Jakub Luptak, Stuart Bloom, Elizabeth Soyode, Martin Besser, Rainer Doffinger, Helen Lee, Gabriel Hawthorne, Stephen MacDonald, Jakub Letowski, and Sara Lear. pCAGGS_SARS-CoV-2_Spike was obtained by the Centre for AIDS Reagents (CFAR), National Institute for Biological Standards and Control (NIBSC), thanks to the donation of Dr. Emma Bentley. R.K.G. is supported by a Wellcome Trust Senior Fellowship in Clinical Science (WT108082AIA). I.G. is supported by a Wellcome Trust Senior Fellowship (207498/Z/17/Z). D.C. is supported by a Wellcome Trust Clinical PhD Research Fellowship. This research was supported by the National Institute for Health Research (NIHR) Cambridge Biomedical Research Centre and the Cambridge Clinical Trials Unit (CCTU). L.C.J. is supported by the Medical Research Council (MRC) (UK; U105181010) and a Wellcome Investigator Award. J.A.G.B. is supported by the European Research Council (ERC) under the European Union's Horizon 2020 research and innovation programme (ERC-CoG-648432 MEMBRANEFUSION) and the MRC (MC_UP_1201/16).

AUTHOR CONTRIBUTIONS

Conceptualization, R.K.G., P.M., D.C., E.G.-K., A.B., and K.G.C.S. Methodology, R.K.G., P.M., D.C., A.R., S.M.A., M.H., I.G., N.T., L.K., L.C.J., K.A.C., X.X., J.A.G.B., J.A.N., R.S., J.P.A. Investigation & Data Collection, R.K.G., P.M., D.C., A.R., S.M.A., M.H., I.G., N.G., B.M., K.C., F.M., V.M., H.Z., L.B., P.B., N.D., P.A.L., J.B., S.B., J.P.A., R.B., M.W., D.S., G.A., and S.H. Writing – Original Draft, R.K.G., P.M., and D.C. Writing – Review & Editing, A.R., S.M.A., E.G.K., A.B., and H.Z.

DECLARATION OF INTERESTS

The authors declare no competing interests.

Received: June 25, 2020
Revised: August 5, 2020
Accepted: August 27, 2020
Published: September 1, 2020

REFERENCES

1. Dong, E., Du, H., and Gardner, L. (2020). An interactive web-based dashboard to track COVID-19 in real time. Lancet Infect. Dis. *20*, 533–534.
2. Collier, D.A., Assennato, S.M., Warne, B., Sithole, N., Sharrocks, K., Ritchie, A., Ravji, P., Routledge, M., Sparkes, D., Skittrall, J., et al. (2020). Point of Care Nucleic Acid Testing for SARS-CoV-2 in Hospitalized Patients: A Clinical Validation Trial and Implementation Study. Cell Rep. Med. *1*, 100062.
3. Arevalo-Rodriguez, I., Buitrago-Garcia, D., Simancas-Racines, D., Zambrano-Achig, P., del Campo, R., Ciapponi, A., Sued, O., Martinez-Garcia, L., Rutjes, L., Low, N., et al. (2020). False-negative results of initial RT-PCR assays for COVID-19: a systematic review. medRxiv. https://doi.org/10.1101/2020.04.16.20066787.
4. Wang, W., Xu, Y., Gao, R., Lu, R., Han, K., Wu, G., and Tan, W. (2020). Detection of SARS-CoV-2 in Different Types of Clinical Specimens. JAMA *323*, 1843–1844.
5. Fang, Y., Zhang, H., Xie, J., Lin, M., Ying, L., Pang, P., and Ji, W. (2020). Sensitivity of Chest CT for COVID-19: Comparison to RT-PCR. Radiology *296*, E115–E117.
6. Tang, Y.W., Schmitz, J.E., Persing, D.H., and Stratton, C.W. (2020). The Laboratory Diagnosis of COVID-19 Infection: Current Issues and Challenges. J. Clin. Microbiol. *58*, e00512–e00520.
7. Arons, M.M., Hatfield, K.M., Reddy, S.C., Kimball, A., James, A., Jacobs, J.R., Taylor, J., Spicer, K., Bardossy, A.C., Oakley, L.P., et al.; Public Health–Seattle and King County and CDC COVID-19 Investigation Team (2020). Presymptomatic SARS-CoV-2 Infections and Transmission in a Skilled Nursing Facility. N. Engl. J. Med. *382*, 2081–2090.
8. He, X., Lau, E.H.Y., Wu, P., Deng, X., Wang, J., Hao, X., Lau, Y.C., Wong, J.Y., Guan, Y., Tan, X., et al. (2020). Temporal dynamics in viral shedding and transmissibility of COVID-19. Nat. Med. *26*, 672–675.
9. Lescure, F.X., Bouadma, L., Nguyen, D., Parisey, M., Wicky, P.H., Behillil, S., Gaymard, A., Bouscambert-Duchamp, M., Donati, F., Le Hingrat, Q., et al. (2020). Clinical and virological data of the first cases of COVID-19 in Europe: a case series. Lancet Infect. Dis. *20*, 697–706.
10. Wölfel, R., Corman, V.M., Guggemos, W., Seilmaier, M., Zange, S., Müller, M.A., Niemeyer, D., Jones, T.C., Vollmar, P., Rothe, C., et al. (2020). Virological assessment of hospitalized patients with COVID-2019. Nature *581*, 465–469.
11. Siddiqi, H.K., and Mehra, M.R. (2020). COVID-19 illness in native and immunosuppressed states: A clinical-therapeutic staging proposal. J. Heart Lung Transplant. *39*, 405–407.
12. Long, Q.X., Liu, B.Z., Deng, H.J., Wu, G.C., Deng, K., Chen, Y.K., Liao, P., Qiu, J.F., Lin, Y., Cai, X.F., et al. (2020). Antibody responses to SARS-CoV-2 in patients with COVID-19. Nat. Med. *26*, 845–848.
13. Suthar, M.S., Zimmerman, M.G., Kauffman, R.C., Mantus, G., Linderman, S.L., Hudson, W.H., Vanderheiden, A., Nyhoff, L., Davis, C.W., Adekunle, O., et al. (2020). Rapid generation of neutralizing antibody responses in COVID-19 patients. Cell Rep. Med. *1*, 100040.
14. Liu, L., Liu, W., Zheng, Y., Jiang, X., Kou, G., Ding, J., Wang, Q., Huang, Q., Ding, Y., Ni, W., et al. (2020). A preliminary study on serological assay for severe acute respiratory syndrome coronavirus 2 (SARS-CoV-2) in 238 admitted hospital patients. Microbes Infect. *22*, 206–211.
15. Lassaunière, R., Frische, A., Harboe, Z.B., Nielsen, A.C.Y., Fomsgaard, A., Krogfelt, K.A., and Jørgensen, C.S. (2020). Evaluation of nine commercial SARS-CoV-2 immunoassays. medRxiv. https://doi.org/10.1101/2020.04.09.20056325.
16. Pickering, S., Betancor, G., Pedro Galao, R., Merrick, B., Signell, A.W., Wilson, H.D., Tan, Kia, Ik, M., Seow, J., Graham, C., Acorset, S., et al. (2020). Comparative assessment of multiple COVID-19 serological technologies supports continued evaluation of point-of-care lateral flow assays in hospital and community healthcare settings. medRxiv. https://doi.org/10.1101/2020.06.02.20120345.

17. Whitman, J.D., Hiatt, J., Mowery, C.T., Shy, B.R., Yu, R., Yamamoto, T.N., Rathore, U., Goldgof, G.M., Whitty, C., Woo, J.M., et al. (2020). Test performance evaluation of SARS-CoV-2 serological assays. medRxiv. https://doi.org/10.1101/2020.04.25.20074856.

18. Collier, D.A., Assennato, S.M., Warne, B., Sithole, N., Sharrocks, K., Ritchie, A., Ravji, P., Routledge, D., Sparkes, D., Skittrall, J., et al. (2020). Point of Care Nucleic Acid Testing for SARS-CoV-2 in Hospitalized Patients: A Clinical Validation Trial and Implementation Study. Cell Rep Med. *1*, 100062.

19. Adams, E.R., Ainsworth, M., Anand, R., Andersson, M.I., Auckland, K., Baillie, J.K., Barnes, E., Beer, S., Bell, J., Berry, T., et al. (2020). Antibody testing for COVID-19: a report from the National COVID Scientific Advisory Panel. medRxiv. https://doi.org/10.1101/2020.04.15.20066407.

20. Korber, B., Fischer, W.M., Gnanakaran, S., Yoon, H., Theiler, J., Abfalterer, W., Hengartner, N., Giorgi, E.E., Bhattacharya, T., Foley, B., et al.; Sheffield COVID-19 Genomics Group (2020). Tracking Changes in SARS-CoV-2 Spike: Evidence that D614G Increases Infectivity of the COVID-19 Virus. Cell *182*, 812–827.e19.

21. Walls, A.C., Park, Y.J., Tortorici, M.A., Wall, A., McGuire, A.T., and Veesler, D. (2020). Structure, Function, and Antigenicity of the SARS-CoV-2 Spike Glycoprotein. Cell *181*, 281–292.e6.

22. Amanat, F., Stadlbauer, D., Strohmeier, S., Nguyen, T.H.O., Chromikova, V., McMahon, M., Jiang, K., Arunkumar, G.A., Jurczyszak, D., Polanco, J., et al. (2020). A serological assay to detect SARS-CoV-2 seroconversion in humans. Nat. Med. *26*, 1033–1036.

23. Gupta, A., Madhavan, M.V., Sehgal, K., Nair, N., Mahajan, S., Sehrawat, T.S., Bikdeli, B., Ahluwalia, N., Ausiello, J.C., Wan, E.Y., et al. (2020). Extrapulmonary manifestations of COVID-19. Nat. Med. *26*, 1017–1032.

24. Zhao, J., Yuan, Q., Wang, H., Liu, W., Liao, X., Su, Y., Wang, X., Yuan, J., Li, T., Li, J., et al. (2020). Antibody responses to SARS-CoV-2 in patients of novel coronavirus disease 2019. Clin. Infect. Dis., ciaa344.

25. Peeling, R.W., Wedderburn, C.J., Garcia, P.J., Boeras, D., Fongwen, N., Nkengasong, J., Sall, A., Tanuri, A., and Heymann, D.L. (2020). Serology testing in the COVID-19 pandemic response. Lancet Infect. Dis *20*, e245–e249.

26. Woo, P.C., Lau, S.K., Wong, B.H., Chan, K.H., Hui, W.T., Kwan, G.S., Peiris, J.S., Couch, R.B., and Yuen, K.Y. (2004). False-positive results in a recombinant severe acute respiratory syndrome-associated coronavirus (SARS-CoV) nucleocapsid enzyme-linked immunosorbent assay due to HCoV-OC43 and HCoV-229E rectified by Western blotting with recombinant SARS-CoV spike polypeptide. J. Clin. Microbiol. *42*, 5885–5888.

27. Jääskeläinen, A.J., Kekäläinen, E., Kallio-Kokko, H., Mannonen, L., Kortela, E., Vapalahti, O., Kurkela, S., and Lappalainen, M. (2020). Evaluation of commercial and automated SARS-CoV-2 IgG and IgA ELISAs using coronavirus disease (COVID-19) patient samples. Euro Surveill. *25*, 2000603.

28. Verdoni, L., Mazza, A., Gervasoni, A., Martelli, L., Ruggeri, M., Ciuffreda, M., Bonanomi, E., and D'Antiga, L. (2020). An outbreak of severe Kawasaki-like disease at the Italian epicentre of the SARS-CoV-2 epidemic: an observational cohort study. Lancet *395*, 1771–1778.

29. Sweeney, N., Merrick, B., Pickering, S., Galao, R.P., Botgros, A., Wilson, H.D., Signell, A.W., Betancor, G., Kia Ik Tan, M., Ramble, J., et al. (2020). Clinical utility of targeted SARS-CoV-2 serology testing to aid the diagnosis and management of suspected missed, late or post-COVID-19 infection syndromes: results from a pilot service. medRxiv. https://doi.org/10.1101/2020.07.10.20150540.

30. Wang, P., Liu, L., Nair, M.S., Yin, M.T., Luo, Y., Wang, Q., Yuan, T., Mori, K., Guzman Solis, A., Yamashita, M., et al. (2020). SARS-CoV-2 Neutralizing Antibody Responses Are More Robust in Patients with Severe Disease. bioRxiv. https://doi.org/10.1101/2020.06.13.150250.

31. Assennato, S.M., Ritchie, A.V., Nadala, C., Goel, N., Zhang, H., Datir, R., Gupta, R.K., Curran, M.D., and Lee, H.H. (2020). Performance evaluation of the point-of-care SAMBA II SARS-CoV-2 Test for detection of SARS-CoV-2. medRxiv. https://doi.org/10.1101/2020.05.24.20100990.

32. Naldini, L., Blömer, U., Gage, F.H., Trono, D., and Verma, I.M. (1996). Efficient transfer, integration, and sustained long-term expression of the transgene in adult rat brains injected with a lentiviral vector. Proc. Natl. Acad. Sci. USA *93*, 11382–11388.

33. Gupta, R.K., Kohli, A., McCormick, A.L., Towers, G.J., Pillay, D., and Parry, C.M. (2010). Full-length HIV-1 Gag determines protease inhibitor susceptibility within in vitro assays. AIDS *24*, 1651–1655.

34. Schmidt, F., Weisblum, Y., Muecksch, F., Hoffmann, H.-H., Michailidis, E., Lorenzi, J.C.C., Mendoza, P., Rutkowska, M., Bednarski, E., Gaebler, C., et al. (2020). Measuring SARS-CoV-2 neutralizing antibody activity using pseudotyped and chimeric viruses. bioRxiv. https://doi.org/10.1101/2020.06.08.140871.

35. Meredith, L.W., Hamilton, W.L., Warne, B., Houldcroft, C.J., Hosmillo, M., Jahun, A.S., Curran, M.D., Parmar, S., Caller, L.G., Caddy, S.L., et al. (2020). Rapid implementation of SARS-CoV-2 sequencing to investigate cases of health-care associated COVID-19: a prospective genomic surveillance study. Lancet Infect. Dis., Published online July 14, 2020. S1473-3099(20)30562-4.

36. Oliveiros, J.C. (2015). Venny. An interactive tool for comparing lists with Venn's diagrams. https://bioinfogp.cnb.csic.es/tools/venny/index.html.

37. Xiong, X., Qu, K., Ciazynska, K.A., Hosmillo, M., Carter, A.P., Ebrahimi, S., Ke, Z., Scheres, S.H.W., Bergamaschi, L., Grice, G.L., et al.; CITIID-NIHR COVID-19 BioResource Collaboration (2020). A thermostable, closed SARS-CoV-2 spike protein trimer. Nat. Struct. Mol. Biol. Published online July 31, 2020. https://doi.org/10.1038/s41594-020-0478-5.

38. Sehnal, D., Rose, A., Koca, J., Burley, S., and Velanker, S. (2018). Mol*: Towards a Common Library and Tools for Web Molecular Graphics. https://doi.org/10.2312/molva.20181103.

STAR★METHODS

KEY RESOURCES TABLE

REAGENT or RESOURCE	SOURCE	IDENTIFIER
Antibodies		
Goat anti-human IgG antibody	Sigma	Cat# A0170
Biological Samples		
Participants combined nose and throat swab	This study	N/A
Participants serum	This study	N/A
Chemicals, Peptides, and Recombinant Proteins		
SARS-CoV-2 Spike protein	Laboratory of J. Briggs	Xiong et al.[37]
SARS-CoV-2 N protein	Laboratory of J. Nathan	N/A
Critical Commercial Assays		
SAMBA II SARS-CoV-2 test	Diagnostics for the real World	Cat# 8500-12
SARS-CoV-2 RT-PCR in-house test on was performed on QIAGEN Roto gene platform	QIAGEN	N/A
COVIDIX 20019 SARS-CoV-2 IgG/IgM Test	COVIDIX Healthcare	Cat# ICOV-402
SureScreen SARS-CoV-2 IgG/IgM Test	SureScreen Diagnostics	Cat# COVID19
Deposited Data		
Mapping and structural mapping of D614G was done on S protein structure deposited in PDB	PDB	RCSB PDB: 6VXX.
Sequences of SARS-CoV-2	GISAID EpiCoV™	www.gisaid.org
Experimental Models: Cell Lines		
Expi293 cells	Laboratory of J. Briggs	Xiong et al.[37]
293T	Laboratory of Greg Towers	N/A
Oligonucleotides		
Next generation sequencing 3 primer set	Laboratory of I. Goodfellow	Meredith et al.[35]
Recombinant DNA		
pCAGGS_SARS-CoV-2_Spike	NIBSC	#100976
pCDNAΔ19Spike-HA	Laboratory of P. Lehner	N/A
pCSFLW	Laboratory of G. Towers	N/A
pCAGGS/ACE2	Laboratory of N. Temperton	N/A
pCAGGS/ TPMPSS2	Laboratory of N. Temperton	N/A
Software and Algorithms		
STATA version 13	STATA	https://www.stata.com/order/download-details/
R 2.6.3	The R project	https://www.r-project.org/
Image Lab	Bio-Rad	N/A
GraphPad Prism 8	GraphPad Software	N/A
Venny	Website	https://bioinfogp.cnb.csic.es/tools/venny/

RESOURCE AVAILABILITY

Lead Contact

Further information should be directed to and will be fulfilled by the Lead Contact, Ravindra K. Gupta rkg20@cam.ac.uk.

Materials Availability

This study did not generate new unique reagents.

Data and Code Availability

Raw anonymised data are available from the lead contact without restriction.

EXPERIMENTAL MODEL AND SUBJECT DETAILS

Clinical Study

The study was conducted in two phases; a clinical validation phase followed by an implementation phase. The study participants in phase one were part of the COVIDx trial,[18] a prospective analytical study which compared SAMBA II SARS-CoV-2 point of care test to the standard laboratory RT-PCR test for the detection of SARS-CoV-2 in participants admitted to Cambridge University Hospitals NHS Foundation Trust (CUH) with a possible diagnosis of COVID-19. Consecutive participants were recruited during 12-hour day shifts over a duration of 4 weeks from the 6th of April 2020 to the 2nd of May 2020. We recruited adults (> 16 years old) presenting to the emergency department or acute medical assessment unit as a possible case of COVID-19 infection. This included any adult requiring hospital admission and who was symptomatic of SARS-CoV-2 infection, demonstrated by clinical or radiological findings.[18] 45 participants who had available stored sera were included in this sub-study and underwent further antibody testing. Phase 2, from July 13th to 27th 2020, comprised a service evaluation of clinical practice whereby adults (> 16 years old) presenting to the emergency department or acute medical assessment unit as a possible case of COVID-19 infection were included. This included any adult requiring hospital admission and who was symptomatic of SARS-CoV-2 infection, demonstrated by clinical or radiological findings.

Cell lines

293T cells were cultured in DMEM complete (DMEM supplemented with 100 U/ml penicillin, 0.1 mg/ml streptomycin, and 10% FCS) and maintained at 37°C in %% CO_2.

Ethical approval

COVIDx (NCT04326387) was approved by the East of England - Essex Research Ethics Committee (REC ref: 20/EE/0109**)**. Serum samples were obtained from patients attending Addenbrooke's Hospital with a suspected or confirmed diagnosis of COVID19. Prospective combined point of care testing of suspected COVID-19 cases was done under CUH NHS Trust service evaluation 3163.

METHOD DETAILS

Test methods

NAAT tests

The standard laboratory RT- PCR test, developed by public health England (PHE), targeting the RdRp gene was performed on a combined nose/throat swab. This test has an estimated limit of detection of 320 copies/ml. In parallel, SAMBA II SARS-CoV-2 testing was performed on a combined nose/throat swab and inactivated in a proprietary buffer at the point of sampling. SAMBA II SARS-CoV-2 targets 2 genes- Orf1 and the N genes and uses nucleic acid sequence based amplification to detect SARS-CoV-2 RNA, with limit of detection of 250 copies/ml.[31]

Pseudotype virus preparation

Viral vectors were prepared by transfection of 293T cells by using Fugene HD transfection reagent (Promega) as follows. Confluent 293T cells were transfected with a mixture of 11ul of Fugene HD, 1μg of pCAGGS_SARS-CoV-2_Spike or pCDNAΔ19Spike-HA, 1ug of p8.91 HIV-1 gag-pol expression vector,[32,33] and 1.5μg of pCSFLW (expressing the firefly luciferase reporter gene with the HIV-1 packaging signal). Viral supernatant was collected at 48 and 72h after transfection, filtered through 0.45um filter and stored at −80°C. The 50% tissue culture infectious dose ($TCID_{50}$) of SARS-CoV-2 pseudovirus was determined using Steady-Glo Luciferase assay system (Promega).

Pseudotype neutralisation assay

Spike pseudotype assays have been shown to have similar characteristics as neutralisation testing using fully infectious wild-type SARS-CoV-2.[34] Virus neutralisation assays were performed on 293T cell transiently transfected with ACE2 and TMPRSS2 using SARS-CoV-2 Spike pseudotyped virus expressing luciferase. Pseudovirus was incubated with serial dilution of heat inactivated human serum samples from COVID-19 suspected individuals in duplicates for 1h at 37°C. Virus and cell only controls were also included. Then, freshly trypsinized 293T ACE2/TMPRSS2 expressing cells were added to each well. Following 48h incubation in a 5% CO_2 environment at 37°C, the luminescence was measured using Steady-Glo Luciferase assay system (Promega).

Enzyme-linked immunosorbent assay (ELISA)

We developed an ELISA targeting the SARS-CoV-2 Spike and N proteins. Trimeric spike protein antigen used in the ELISA assays consists of the complete S protein ectodomain with a C-terminal extension containing a TEV protease cleavage site, a T4 trimerization foldon and a hexa-histidine tag. The S1/S2 cleavage site with amino acid sequence PRRAR was replaced with a single Arginine residue and stabilizing Proline mutants were inserted at positions 986 and 987. Spike protein was expressed and purified from Expi293 cells (Thermo Fisher). N protein consisting of residues 45-365 was initially expressed as a His-TEV-SUMO-fusion. After Ni-NTA purification, the tag was removed by TEV proteolysis and the cleaved tagless protein further purified on Heparin and gel filtration columns.

The ELISAs were in a stepwise process; a positivity screen was followed by endpoint titer as previously described.[22] Briefly, 96-well EIA/RIA plates (Corning, Sigma) were coated with PBS or 0.1 μg per well of antigen at 4°C overnight. Coating solution was removed, and wells were blocked with 3% skimmed milk prepared in PBS with 0.1% Tween 20 (PBST) at ambient temperature for 1 hour. Previously inactivated serum samples (56°C for 1 hour) were diluted to 1:60 or serially diluted by 3-fold, six times in 1% skimmed milk in PBST. Blocking solution was aspirated and the diluted sera were added to the plates and incubated for 2 hours at ambient temperature. Diluted sera were removed, and plates were washed three times with PBST. Goat anti-human IgG secondary antibody-Peroxidase (Fc-specific, Sigma) prepared at 1:3,000 in PBST was added and plates were incubated for 1 hour at ambient temperature. Plates were washed three times with PBST. ELISAs were developed using 3,5,3′,5′- tetramethylbenzidine (TMB, ThermoScientific); reactions were stopped after 10 minutes using 0.16M Sulfuric acid.

COVIDIX 2019 SARS-CoV-2 IgG/IgM Test (COVIDIX Healthcare, Cambridge, UK)

This colloidal-gold lateral flow immunoassay is designed to detect IgG and IgM to SARS-CoV-2. The test is CE marked. It was used according to the manufacturer's instructions. 10μl of serum was added to the test well followed by 2 drops of the manufacturer's proprietary buffer. In order to rule out cross reactivity of this test with seasonal coronavirus antibodies we tested 19 stored specimens from before 2020, some of which had N and S protein SARS-CoV-2 cross reactivity (Table S2).

SureScreen SARS-CoV-2 IgG/IgM Test (SureScreen Diagnostics Ltd, Derby, UK)

This colloidal-gold lateral flow immunoassay is designed to detect IgG and IgM to SARS-CoV-2. It was used according to the manufacturer's instructions. The test has been CE marked and previously validated against a large panel of negative historical controls and in serum from confirmed PCR positive COVID-19 cases.[16] 10μl of serum was added to the test well followed by 2 drops of the manufacturer's proprietary buffer.

Next generation sequencing of SARS-CoV-2 isolates in nose/throat swabs

Samples with CT values above 33 were sequenced with a multiplex PCR approach according to the ARTIC version 2 protocol with version 3 primer set. Amplicons were sequenced using MinION flow cells version 9.4.1 (Oxford Nanopore Technologies, Oxford, UK). Genomes were assembled as previously described.[35] The sequences are freely available from GISAID EpiCoV™ under accession IDs: EPI_ISL 433757, 433754, 433792, 433850, 433751, 433778, 433869, 433875, 433874, 433917, 433962, 433956, 434034, 438681, 438711 and 444331. The submitting laboratory is the COVID-19 Genomics UK (COG-UK) Consortium and the originating laboratory is Department of Pathology, University of Cambridge.

QUANTIFICATION AND STATISTICAL ANALYSIS

Enzyme-linked immunosorbent assay (ELISA) quantification

The optical density at 450 nm (OD450) was measured using a Spectramax i3 plate reader. The absorbance values for each sample were determined by subtracting OD values from uncoated wells. All data analyses were performed using Prism 8 version 8.4.2 (GraphPad). An OD cut off of 0.3 was used to define a positive IgG response to full length Spike protein.

COVIDIX 2019 nCoV IgG/IgM Test band density

For quantification of IgG and IgM band density in COVIDIX 2019 nCoV IgG/IgM Test, high resolution images of completed POC antibody test cassettes were acquired using ChemiDoc MP Imaging System (Bio-Rad) at 20min post-addition of the human serum. Band intensities were analyzed using Image Lab software (Bio-Rad).

Quantification of neutralisation sensitivity

The 50% inhibitory dilution (EC_{50}) was defined as the serum dilution at which the relative light units (RLUs) were reduced by 50% compared with the virus control wells (virus + cells) after subtraction of the background RLUs in the control groups with cells only. The EC_{50} values were calculated with non-linear regression, log (inhibitor) versus normalized response using GraphPad Prism 8 (GraphPad Software, Inc., San Diego, CA, USA). The neutralisation assay was positive if the serum achieved at least 50% inhibition at 1 in 3 dilution of the SARS-CoV-2 spike protein pseudotyped virus in the neutralisation assay. The neutralisation result was negative if it failed to achieve 50% inhibition at 1 in 3 dilution.

Assessment of neutralisation assay performance

Four assays detecting IgG to COVID-19 were utilized in this study. 38 of the 45 samples were identified as concordant with at least three of the four assays and considered confirmed either negative or positive. Against this group of samples validated for content of COVID-19 IgG, each individual assay was assessed. Neutralisation, ELISA, SureScreen and COVIDIX assays gave a correct result in 100%, 97.4%, 92.1% and 86.8%, respectively, justifying the choice of the neutralisation assay as standard.

Analyses

The performance of SAMBA II SARS-CoV-2 test and COVIDIX SARS-CoV-2 IgG/IgM Test or SureScreen SARS-CoV-2 IgG/IgM Test for diagnosing COVID-19 were calculated alone and then in combination along with binomial 95% confidence intervals (CI). A composite reference standard was used - standard lab RT-PCR and a neutralisation assay. Descriptive analyses of clinical and

demographic data are presented as median and interquartile range (IQR) when continuous and as frequency and proportion (%) when categorical. The differences in continuous and categorical data were tested using Wilcoxon rank sum and Chi-square test respectively. Statistical analysis were conducted using Stata (version 13) and GraphPad Prism (version 8), with additional plots generated using GraphPad Prism. Venn diagrams were prepared using Venny.[36] Structural modeling of location of D614G was done using Mol*.[38]

ADDITIONAL RESOURCES

COVIDx was registered with the ClinicalTrials.gov Identifier NCT04326387.

Cell Reports Medicine, Volume *1*

Supplemental Information

Combined Point-of-Care Nucleic Acid and Antibody Testing for SARS-CoV-2 following Emergence of D614G Spike Variant

Petra Mlcochova, Dami Collier, Allyson Ritchie, Sonny M. Assennato, Myra Hosmillo, Neha Goel, Bo Meng, Krishna Chatterjee, Vivien Mendoza, Nigel Temperton, Leo Kiss, Leo C. James, Katarzyna A. Ciazynska, Xiaoli Xiong, John A.G. Briggs, James A. Nathan, Federica Mescia, Laura Bergamaschi, Hongyi Zhang, Petros Barmpounakis, Nikos Demeris, Richard Skells, Paul A. Lyons, John Bradley, Steven Baker, Jean Pierre Allain, Kenneth G.C. Smith, Rachel Bousfield, Michael Wilson, Dominic Sparkes, Glenn Amoroso, Effrosyni Gkrania-Klotsas, Susie Hardwick, Adrian Boyle, Ian Goodfellow, Ravindra K. Gupta, and The Cambridge Institute of Therapeutic Immunology and Infectious Disease-National Institute of Health Research (CITIID-NIHR) COVID BioResource Collaboration

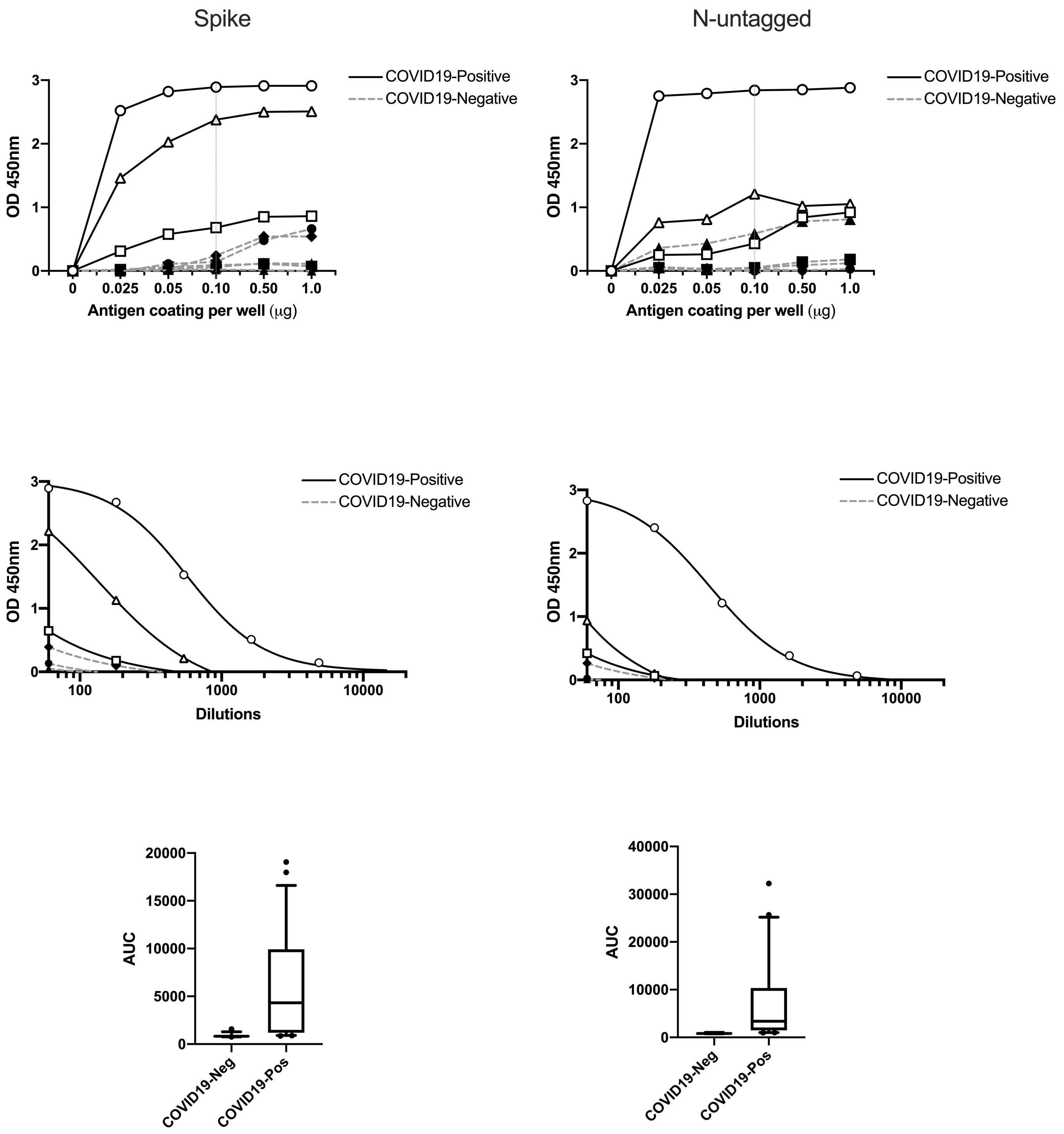

Supplementary Figure 1, related to Figure 1: Establishment of serological assay to determine positivity and endpoint titre against human SARS CoV-2. Residual stored serum samples from PCR positive and negative patient cohort were screened for reactivity against full-length spike and N-proteins. A) To determine the appropriate concentration of antigen used for plate coating, 0, 0.025, 0.05, 0.1, 0.5 and 1.0 1mg antigen per well was coated and reactivity of known seropositive and seronegative serum samples were examined. B) Subsequently, end-point titrations were performed using 0.1mg per well spike and N antigen coating. C. The area under the curve (AUC) was calculated for every sample using end point titrations against spike (n=76) and N protein (n=64), and the mean and the 95% confidence intervals are shown for all PCR positive and negative samples. OD: optical density (nanometers)

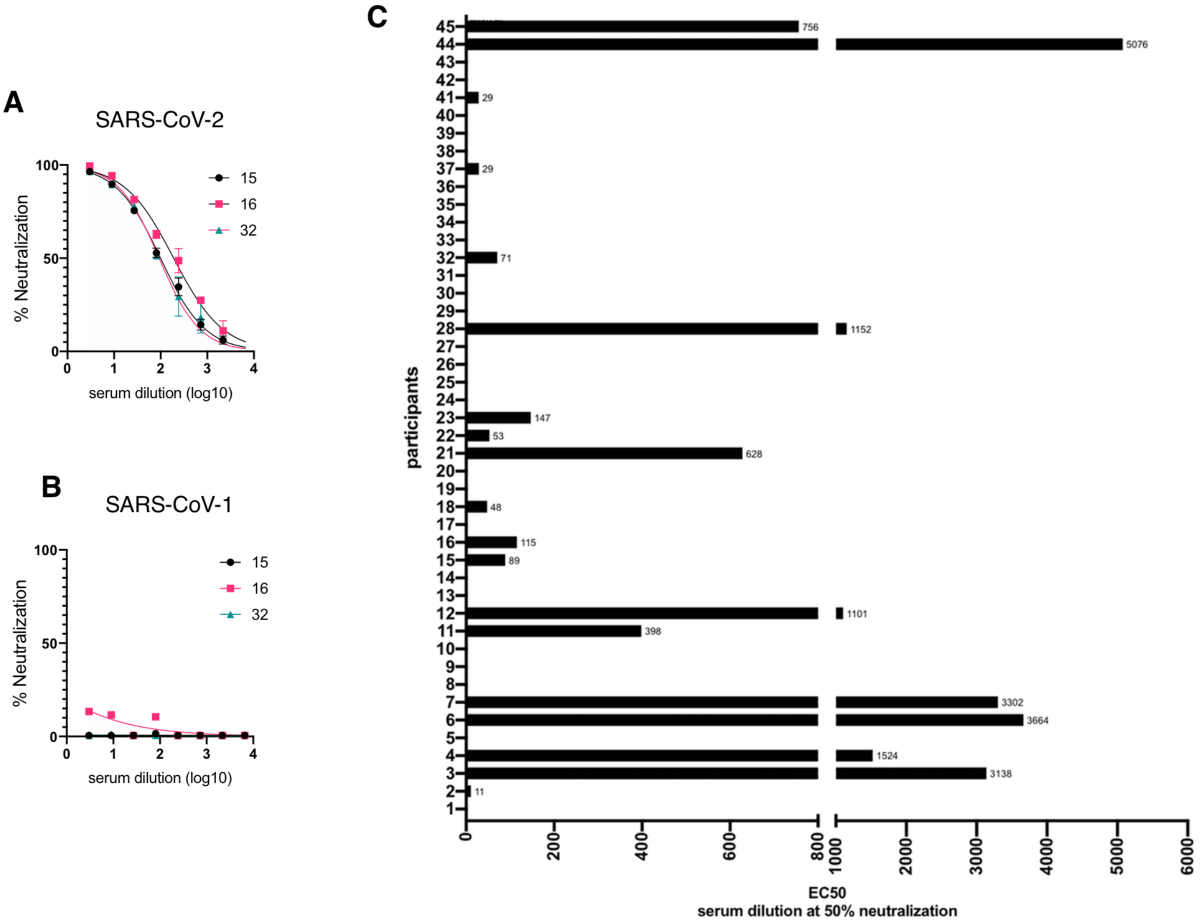

Supplementary Figure 2, related to Figure 1 : Specificity of antibody neutralizing response against SARS-CoV-2 and CoV-1.

SARS-CoV-2 **(A)** or SARS-CoV-1 **(B)** Spike protein pseudotyped viral particles were incubated with serial dilutions of heat inactivated human serum samples from Covid-19 suspected individuals (#15,16,32) in duplicates for 1h at 37˚C. 293T ACE2/TMPRSS2 expressing cells were added to each well. Following 48h incubation in a 5% CO2 environment at 37°C, the luminescence was measured using Steady-Glo Luciferase assay system (Promega). Percentage of neutralization was calculated with non-linear regression, log (inhibitor) vs. normalized response using GraphPad Prism 8 (GraphPad Software, Inc., San Diego, CA, USA). **(C)** The 50% inhibitory dilution (EC50) was defined as the serum dilution at which the relative light units (RLUs) were reduced by 50% compared with the virus control wells (virus + cells) after subtraction of the background RLUs in the control groups with cells only. The EC50 values were calculated with non-linear regression, log (inhibitor) vs. normalized response using GraphPad Prism 8 (GraphPad Software, Inc., San Diego, CA, USA).

A

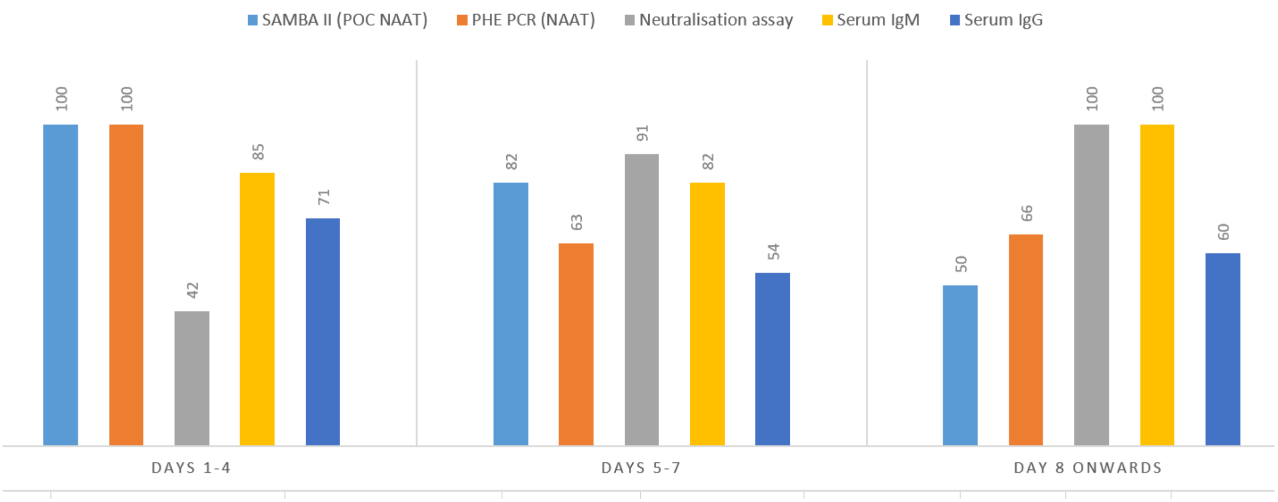

B

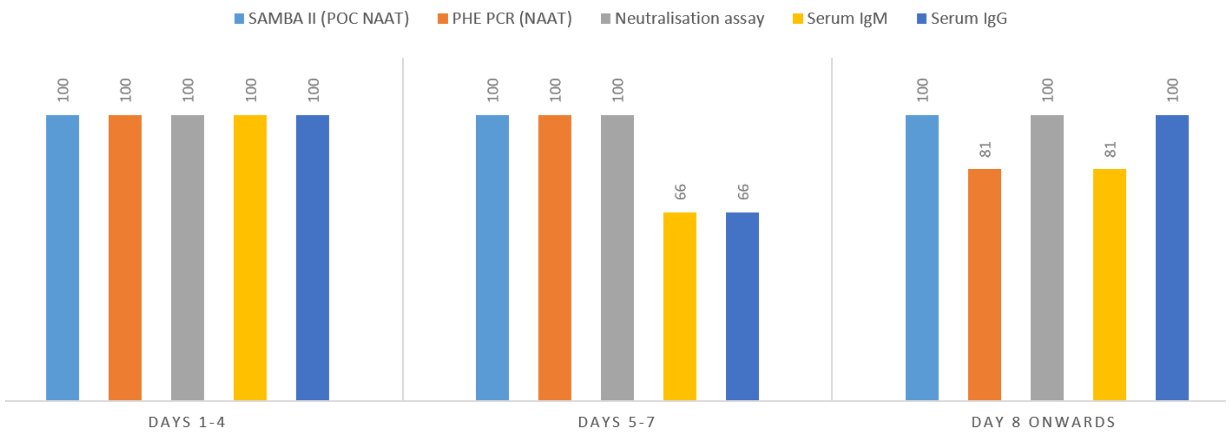

Supplementary Figure 3:, related to Figure 1. Results of assays by time since onset of symptoms (A) % of positive tests in individuals classified as COVID-19 positive by composite reference (B) % of negative tests in individuals classified as COVID-19 negative by composite reference. Here the serum IgM and IgG assay is COVIDIX SARS-CoV-2 IgM/IgG test.

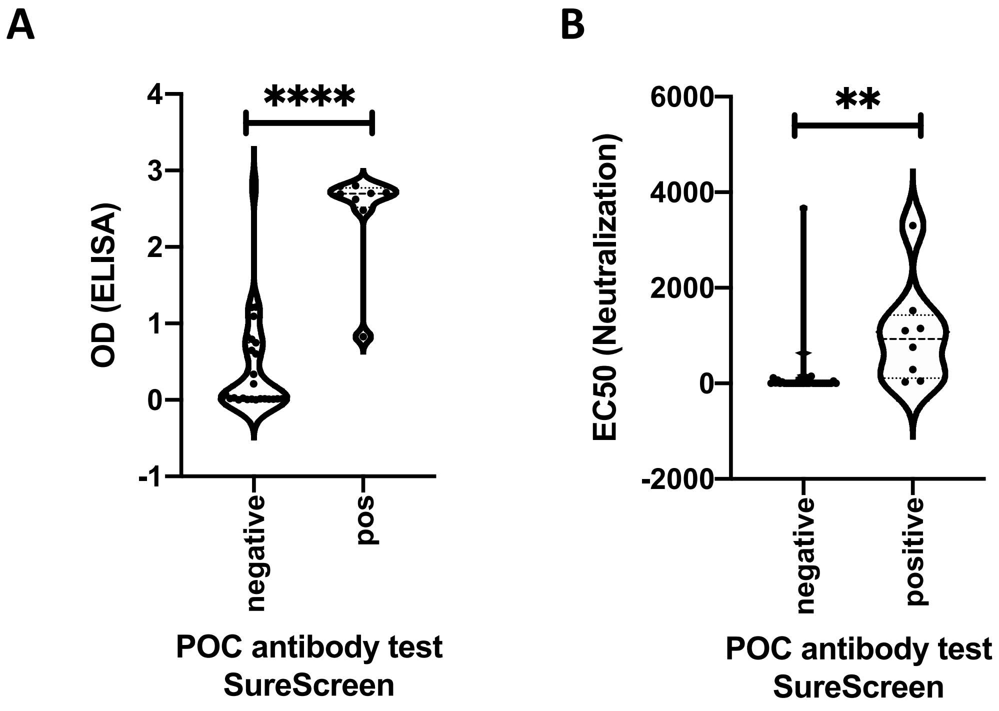

Supplementary Figure 4, related to Figure 1: Comparison of a lateral flow diagnostic test (SureScreen SARS-CoV-2 IgM/IgG test) against ELISA IgG and SARS-CoV-2 pseudotyped virus neutralisation assays on sera from patients with suspected moderate to severe COVID-19. (A) Comparison between ELISA IgG and positive/negative POC IgG band results for SureScreen SARS-CoV-2 IgM/IgG test. n=38, p<0.0001. (B). Comparison between EC50 dilution titre from neutralisation assay and positive/negative SureScreen SARS-CoV-2 IgM/IgG antibody band test results. n=43, p=0.005.

Supplementary Table 1, relates to Figure 1: Pre 2020 sera testing: ELISA optical density values for full length SARS-CoV-2 Spike (FL), Spike receptor binding domain (RBD), nucleocapsid (N), and result on testing with COVIDIX SARS-CoV-2 IgM/IgG test. Positive (from confirmed positive) and negative (pooled human sera from pre 2020) control values are given.

Sample no	FL	RBD	N	COVIDIX IgM/IgG result
1	0.95735	0.20455	0.5343	Negative
2	0.1217	0.1008	0.0746	Negative
3	0.2680	0.1300	0.1285	Negative
4	0.2511	0.0837	0.07445	Negative
5	0.10625	0.06625	0.4722	Negative
6	0.1561	0.08655	0.0927	Negative
7	1.12375	0.05785	0.40535	Negative
8	0.1432	0.0888	0.5842	Negative
9	0.49075	0.06505	0.32445	Negative
10	0.16075	0.03625	0.13485	Negative
11	0.08205	0.0504	0.07485	Negative
12	0.1956	0.23025	0.1748	Negative
13	0.1482	0.07115	0.05645	Negative
14	0.16075	0.078	1.00845	Negative
15	0.18015	0.09845	0.7598	Negative
16	0.26335	0.0693	0.38865	Negative
17	0.1864	0.18905	0.35065	Negative
18	0.1265	0.3684	0.18025	Negative
19	0.08425	0.06555	0.1378	Negative
Negative	0.297	0.054	0.387	
Positive	2.704	2.150	2.337	

Supplementary table 2, relates to Figure 3: Characteristics of 16 COVID-19 positive participants with available SARS-CoV-2 sequence data differentiated by D614G Spike mutation.

Amino acid at 614	Days post symptom onset	NAAT result	Serum Neutralisation assay	Spike ELISA	COVIDIX IgM/IgG	Sure Screen
D	7	Positive	Negative	Positive	Positive	Positive
D	7	Positive	Negative	Negative	Negative	Negative
G	1	Positive	Negative	Positive	Positive	Negative
G	11	Positive	Positive	Positive	Positive	Positive
G	7	Positive	Positive	Positive	Positive	Positive
G	1	Positive	Negative	Negative	Positive	Negative
G	9	Positive	Positive	Positive	Positive	Positive
G	21	Positive	Positive	Positive	Positive	Positive
G	1	Positive	Positive	Positive	Positive	Positive
G	1	Positive	Positive	Positive	Positive	Positive
G	7	Positive	Positive	Positive	Positive	Positive
G	7	Positive	Positive	Positive	Positive	Positive
G	1	Positive	Negative	Negative	Positive	Negative
G	1	Positive	Negative	Positive	Positive	Negative
G	1	Positive	Positive	Positive	Positive	Positive
G	6	Positive	Positive	Positive	Positive	Positive
